# A new carbohydrate-active oligosaccharide dehydratase is involved in the degradation of ulvan

**DOI:** 10.1016/j.jbc.2021.101210

**Published:** 2021-09-20

**Authors:** Marcus Bäumgen, Theresa Dutschei, Daniel Bartosik, Christoph Suster, Lukas Reisky, Nadine Gerlach, Christian Stanetty, Marko D. Mihovilovic, Thomas Schweder, Jan-Hendrik Hehemann, Uwe T. Bornscheuer

**Affiliations:** 1 Department of Biotechnology & Enzyme Catalysis, Institute of Biochemistry, University Greifswald, Greifswald, Germany; 2 Department of Pharmaceutical Biotechnology, Institute of Pharmacy, University Greifswald, Greifswald, Germany; 3 Institute of Applied Synthetic Chemistry, TU Wien, Vienna, Austria; 4 Max Planck-Institute for Marine Microbiology, Bremen, Germany; 5 Center for Marine Environmental Sciences (MARUM), University of Bremen, Bremen, Germany

**Keywords:** pathway elucidation, carbohydrate-active enzymes, novel enzyme, dehydratase, marine polysaccharide, ulvan, enzyme mechanism, sulfatase, glycoside hydrolase, C-PAGE, carbohydrate electrophoresis, CAZyme, carbohydrate active enzyme, DHy, Dehydratase, FACE, fluorophore-assisted carbohydrate electrophoresis, GH, glycoside hydrolase, PL, polysaccharide lyase, PUL, polysaccharide utilization loci

## Abstract

Marine algae catalyze half of all global photosynthetic production of carbohydrates. Owing to their fast growth rates, *Ulva* spp. rapidly produce substantial amounts of carbohydrate-rich biomass and represent an emerging renewable energy and carbon resource. Their major cell wall polysaccharide is the anionic carbohydrate ulvan. Here, we describe a new enzymatic degradation pathway of the marine bacterium *Formosa agariphila* for ulvan oligosaccharides involving unsaturated uronic acid at the nonreducing end linked to rhamnose-3-sulfate and glucuronic or iduronic acid (Δ-Rha3S-GlcA/IdoA-Rha3S). Notably, we discovered a new dehydratase (P29_PDnc) acting on the nonreducing end of ulvan oligosaccharides, *i.e.*, GlcA/IdoA-Rha3S, forming the aforementioned unsaturated uronic acid residue. This residue represents the substrate for GH105 glycoside hydrolases, which complements the enzymatic degradation pathway including one ulvan lyase, one multimodular sulfatase, three glycoside hydrolases, and the dehydratase P29_PDnc, the latter being described for the first time. Our research thus shows that the oligosaccharide dehydratase is involved in the degradation of carboxylated polysaccharides into monosaccharides.

Marine algae catalyze half of the global photosynthetic production of carbohydrates (1). Fast growth makes macroalgae a promising renewable bioresource for the chemical, pharmaceutical, agricultural, and food industry (2, 3, 4, 5, 6, 7, 8). Exemplarily, the ubiquitous green seaweed *Ulva* spp. has been recently suggested as a source of bioactive and rare sugars (9, 10). Ulvan, which is branched and highly sulfated, is the major cell wall polysaccharide of the ‘green tide’ causing macroalgae *Ulva* spp. Ulvan can represent up to 30% of the algal dry weight (2). The major disaccharide repeating units are ulvanobiouronic acid A (β-d-glucuronic acid (GlcA)-(1,4)-α-l-rhamnose-3-sulfate (Rha3S)), ulvanobiouronic acid B (α-l-iduronic acid (IdoA)-(1,4)-α-l-rhamnose-3-sulfate), ulvanobiose-3-sulfate (β-d-xylose (Xyl)-(1,4)-α-l-rhamnose-3-sulfate), and ulvanobiose-2′,3-disulfate (β-d-xylose-2-sulfate (Xyl2S)-(1,4)-α-l-rhamnose-3-sulfate). A modification of Rha3S by β-1,2-linked GlcA side chains and the appearance of consecutive GlcA residues have been described as well (2).

In polysaccharide degrading bacteria, the genes encoding for the carbohydrate-active enzymes (CAZymes) often colocalize with transporter genes in gene clusters called ‘polysaccharide utilization loci’ (PULs) (11) as it is the case for the ulvan pathway of the marine *Flavobacterium Formosa agariphila* KMM 3901T (12). Recently, we elucidated the degradation cascade for ulvan consisting of 12 carbohydrate-active enzymes, including two polysaccharide lyases, three sulfatases, and seven glycoside hydrolases (10). This pathway enables the degradation of ulvan into monosaccharides.

Polysaccharide lysases (PL) of the families PL24, PL25, PL28, and PL40 catalyze the initial degradation step of ulvan into oligosaccharides *via* an elimination mechanism. This mechanism forms an unsaturated uronic acid residue at the nonreducing end (10, 13, 14, 15, 16, 17, 18, 19), which then can be cleaved by unsaturated glucuronyl hydrolases of the families GH88 or GH105 (2, 10, 20, 21). Importantly, this unsaturated sugar is required for the activity of GH105 and GH88. In the abovementioned pathway, there are also glycoside hydrolases of family GH78 (P36_GH78) that lead to the formation of a nonreducing end with a saturated uronic acid sugar. This one can only be cleaved by a GH3 family enzyme.

Here, we describe the discovery of a new class of ulvan-active dehydratases. This new enzyme type converts saturated uronic acid sugars such as GlcA/IdoA-Rha3S at the nonreducing end of oligosaccharides into unsaturated sugars enabling cleavage by GH105. This is the first time a dehydratase was reported as an enzyme active on carbohydrates.

## Results

**Figure 1:**
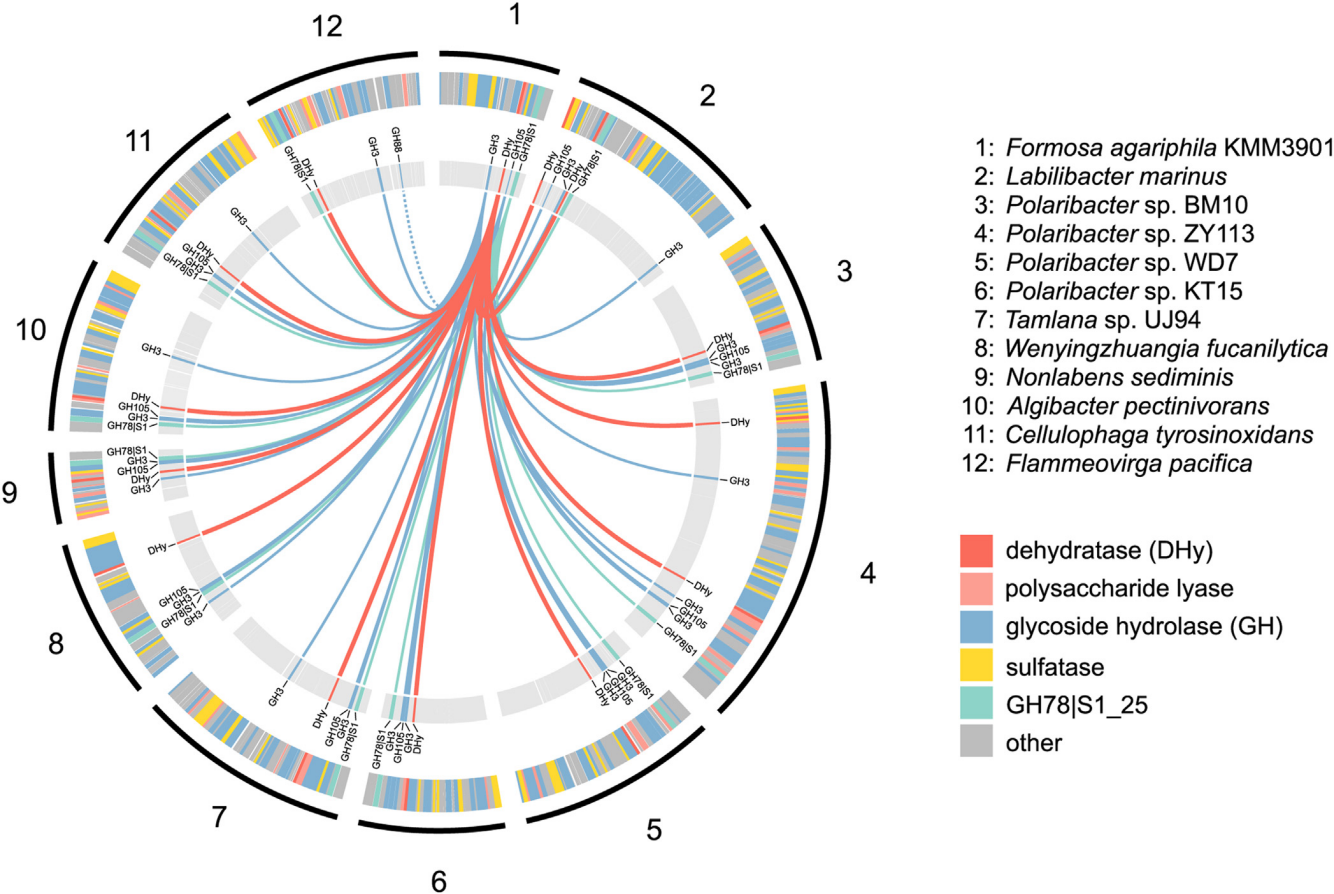
**Genomic overview of putative ulvan PULs containing the alternative pathway analogues genes in 12 marine Bacteroidetes.** Ulvan PUL annotations are given in the *outer ring*, whereas alternative pathway analogues genes for CAZymes and the GH78|S1_25 hybrid are highlighted within the *inner ring*, linked to the model organism of this study, *F. agariphila* KMM 3901. The *dashed line* indicates the closely related GH88 family of GH105.

The initial degradation step catalyzed by the ulvan lyases (P10_PLnc and P30_PL28) leads to the formation of several oligosaccharides with diverse composition, see also Fig. S1 (10). This includes glucuronic or iduronic acid containing tetramers, which are the result of an incomplete digestion. Even though these oligosaccharides can be degraded by the ulvan lyases to the dimer Δ-Rha3S (10, 18), the oligosaccharides Δ-Rha3S-GlcA-Rha3S and Δ-Rha3S-IdoA-Rha3S accumulated when ulvan was degraded, because high concentrations of lyase products inhibit the reaction (22). This was our motivation to search for suitable enzyme activities within PUL H of *F. agariphila* that support the conversion of these intermediates. Comparative genomics of 12 bacteroidetal ulvan PULs revealed the presence of a conserved unknown enzyme, P29_PDnc, in *F. agariphila* KMM 3901, which showed an induced expression during cultivation with ulvan as sole carbon source (Fig. 1) (10).

**Figure 2:**
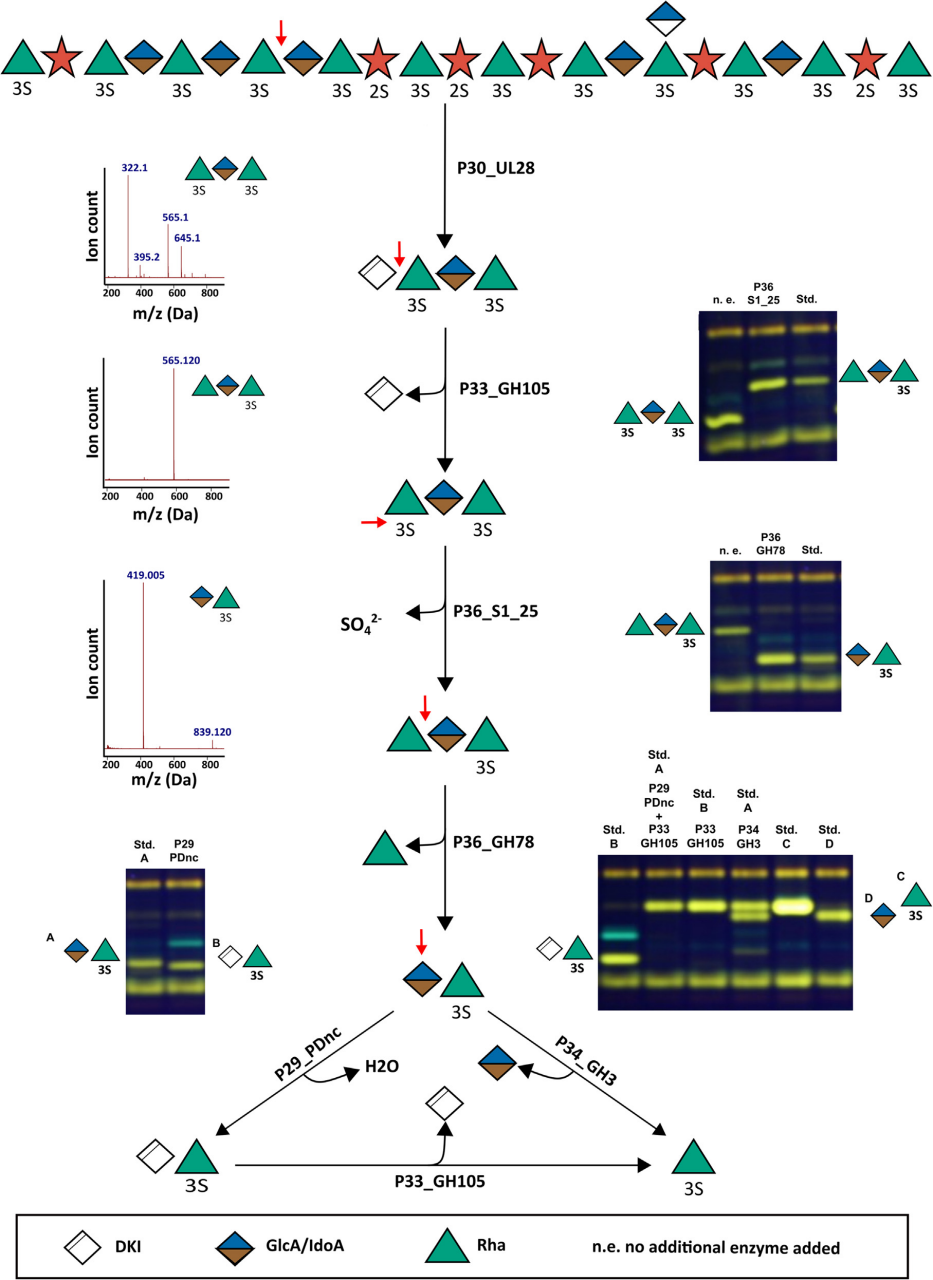
**Model of the alternative ulvan degradation pathway of *F. agariphila* based on FACE analyses and MS data.** The activity of the P33_GH105 to cleave 4-deoxy-α-l-threo-hex-4-enopyranuronic acid from the tetramer Δ-Rha3S-GlcA/IdoA-Rha3S to the trimers Rha3S-GlcA/IdoA-Rha3S was previously described by Reisky *et al.* (10). In reactions containing P29_PDnc, the other enzymes were heat-inactivated before addition of P29_PDnc, to prevent a degradation of the dehydratase product by P33_GH105. All products and standards (except GlcA) used had been isolated and confirmed by MS and NMR measurement. The MS data were derived from the HPLC−ELS-MS measurement from the purified oligomers from the natural product. The last lane of the *top gel* is the same as the first lane of the *middle gel*, and the last lane of the *middle gel* is the same as the first lane of the *left gel* while the *right lane* of the *left gel* is the first lane of the last *right gel* to ensure continuity in the explanation of the degradation pathway. The full FACE-Gel picture can be found in the Supporting information (Fig. S20). The standard for GlcA was obtained from the company Carl-Roth. All products represent the mixture of both oligomers containing one of the epimers GlcA or IdoA. The ratio between GlcA- and IdoA-containing oligomers is ∼70:30 (18). The 4-deoxy-α-l-threo-hex-4-enopyranuronic acid is abbreviated with “unsaturated uronic acid.”

Biochemical characterization led to the discovery of a new enzymatic function in the degradation of uronic polysaccharides such as ulvan. This enabled the complete degradation of Δ-Rha3S-GlcA-Rha3S and Δ-Rha3S-IdoA-Rha3S (Fig. 2, see also Fig. S1). As it was not possible to separate these two oligosaccharides, this mixture was designated as Δ-Rha3S-GlcA/IdoA-Rha3S. At the first step of the degradation cascade, the *exo*-acting unsaturated glucuronyl hydrolase (P33_GH105) cleaves the unsaturated uronyl residue from the nonreducing end (9, 22, 23). The products of this reaction are 5-dehydro-4-deoxy-d-glucuronate and the trisaccharide Rha3S-GlcA/IdoA-Rha3S. Its chemical structure, which was previously confirmed (10), shows similarity to Rha3S-Xyl-Rha3S and Rha3S-Xyl2S-Rha3S (Table S1 and Figs. S2–S5), which are substrates for the sulfatase domain of P36_S1_25 (10). The P36 is a multidomain protein, which consists of the sulfatase domain S1_25 and an α-l-rhamnosidase domain GH78 (9). Indeed, the sulfatase converts all three products irrespectively of the sugar species located between the two flanking rhamnose residues. In all three cases, it desulfates the rhamnose residue at the nonreducing end so that this sulfatase is active on ulvan trisaccharides with the general structure Rha3S-XXX-Rha3S (10). The desulfated trisaccharide Rha-GlcA/IdoA-Rha3S was isolated confirming the desulfation at the nonreducing end (Table S2 and Figs. S8–S11). Analogous to the already established pathway, Rha-GlcA/IdoA-Rha3S is now degraded by the α-l-rhamnosidase domain of P36_GH78 leading to the removal of the rhamnose residue at the nonreducing end (Table S3 and Figs. S12–S19). This confirms that both enzyme domains of P36 act in consecutive steps within the ulvan degradation on multiple substrate molecules. This makes P36 the first discovered multimodular enzyme participating in ulvan degradation. The carbohydrate structures along the pathway to the reaction product GlcA/IdoA-Rha3S were elucidated by NMR spectroscopy (Tables S1–S3 and Figs. S2–S19). Proceeding from this dimeric intermediate, the pathway splits into two subpathways (Fig. 2), which could be identified by screening all produced enzymes encoded by PUL H. The first way to digest the disaccharide GlcA/IdoA-Rha3S is to use P34_GH3 (Fig. 2). This glycoside hydrolase cleaves the glucuronic or iduronic acid residues with release of rhamnose-3-sulfate, making it a promiscuous β-glucuronidase/α-iduronidase. The second way is to use the conserved hypothetical protein P29_PDnc in the first step (Fig. 2). This enzyme converts the disaccharide GlcA/IdoA-Rha3S into the formerly described disaccharide Δ-Rha3S by elimination of water. The disaccharide Δ-Rha3S can also be produced using the previously described ulvan lyases, see also Fig. S1 (10). P33_GH105 hydrolyzes the formed disaccharide as described before, leading to the formation of Rha3S and 5-dehydro-4-deoxy-d-glucuronate, which confirms the dehydratase activity of P29_PDnc (Fig. 2). Increased abundance of these four CAZymes in ulvan-grown *F. agariphila* KMM 3901 (10), as well as the occurrence in diverse marine Bacteroidetes (Fig. 1), emphasizes the importance of this alternative pathway for the efficient degradation of ulvan.

The two resulting products of both pathways—rhamnose-3-sulfate and glucuronic or iduronic acid—were confirmed by using commercial substrates as standards (Fig. 2). Thereby, P29_PDnc was identified to be a novel type of ulvan-active dehydratase that participates in the degradation of ulvan. This is the first time a dehydratase was described to be acting in the depolymerization of a carbohydrate. Other described sugar-active dehydratases usually catalyze monosaccharide-related reactions (24, 25, 26).

**Figure 3:**
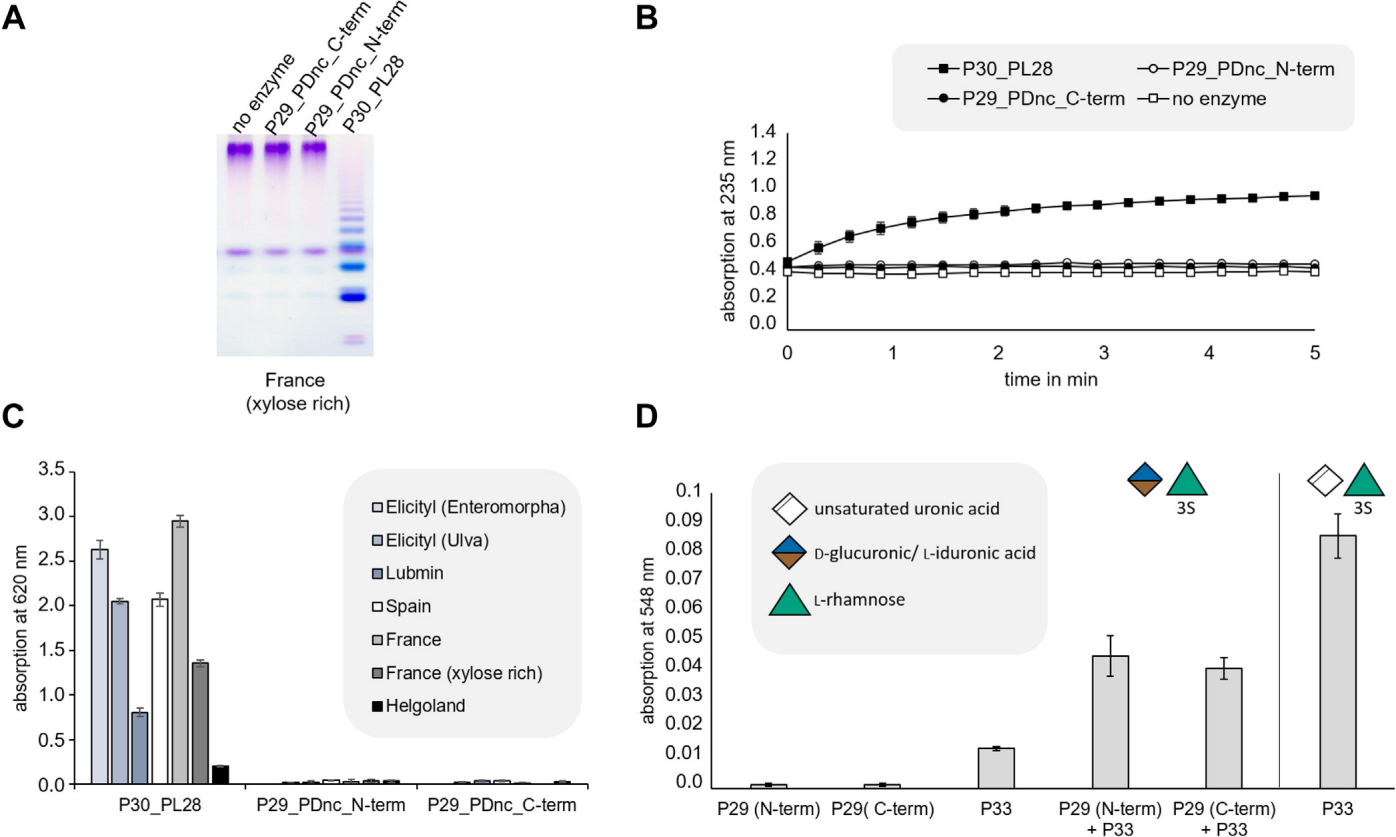
**Analysis of lyase and dehydratase activity of P29_PDnc.***A*, C-PAGE analysis, (*B*) lyase assay with ulvan from France (xylose rich), (*C*) reducing-end assay, and (*D*) thiobarbituric acid assay. Polymeric ulvan from seven different sources has been incubated with both P29_PDnc variants with N- or C-terminal His-tag and P30_PL28 as positive control or without enzymes as negative control. We used in these experiments two commercially available ulvans from Elicityl extracted from *Enteromorpha* sp. or *Ulva* sp., and five self-isolated ulvans from “kulau sea lettuce” containing *Ulva* spp. from Spain, and from self-collected *Ulva* sp. from Helgoland (North Sea), France (Atlantic Ocean) and Lubmin (Baltic Sea) (see Figs. S20–S26). Purified GlcA/IdoA-Rha(3S) was incubated with both P29_PDnc variants with N- or C-terminal His-tag and/or P33_GH105 or purified Δ-Rha(3S) was incubated with P33_GH105 as a positive control. The resulting reaction mixture was investigated using the thiobarbituric acid assay for the determination of 5-dehydro-4-deoxy-d-glucuronate (23).

In previous studies, P29_PDnc was reported to be an ulvan lyase with broad substrate spectrum (25). In contrast, in this work no activity of this enzyme against polymeric ulvan from seven different sources could be observed. One difference between the constructs used in our study and the published example is the position of the His-tag at the investigated heterologous produced enzymes. To investigate if the His-tag position influences the enzymatic activity, two different variants, one with N-terminal and the other with C-terminal His-tag, were prepared. A spectrophotometric lyase assay was used to determine the double bond formation, which is characteristic for the lyase activity (18). Carbohydrate polyacrylamide gel electrophoresis (C-PAGE) was used to visualize the breakdown products, and a reducing-end assay was used to estimate the reducing ends resulting from this cleavage process (Fig. 3, *A*–*C* and Figs. S21–S27). However, we still could not detect lyase activity in any of the C-terminal or N-terminal His-Tag P29_PDnc variants on ulvans from seven different sources, while the positive control P30_PL28 (18) showed activity (Fig. 2 and Figs. S21–S27). However, the P29_PDnc activity against the disaccharide GlcA/IdoA-Rha3S could be confirmed by a thiobarbituric acid assay (23) (Fig. 3*D*). Both C-terminal and N-terminal His-Tag P29_PDnc variants and the supporting enzyme P33_GH105, which releases the 5-dehydro-4-deoxy-d-glucuronate after the dehydratase reaction, were incubated with the target disaccharide. As a positive control P33_GH105 was also used in biocatalysis reactions on the lyase-produced disaccharide Δ-Rha3S, which is the reaction product of the dehydratase reaction. The batches containing only a C-terminal or N-terminal His-Tag variant of P29_PDnc showed no absorption as the P33_GH105, which is supposed to release the formed 5-dehydro-4-deoxy-d-glucuronate, is missing. P33_GH105 on its own induced a very small absorption, but only a combination of P29_PDnc and P33_GH105 led to a significant signal. However, this is far from the absorption values observed for the positive control using P33_GH105 on the disaccharide Δ-Rha3S (Fig. 3*D*). The thiobarbituric acid assay thereby confirms the results obtained by FACE analysis that P29_PDnc converts GlcA/IdoA-Rha3S to Δ-Rha3S, which can be targeted by P33_GH105. As the substrate GlcA/IdoA-Rha3S is converted by both, P29_PDnc and P34_GH3, it was possible to reverse the dehydratase reaction by shifting the equilibrium to the educt side (Fig. S28). When the reaction was carried out in deuterium oxide (D_2_O), the deuterium was inserted at the double bond, which could be identified and confirmed by mass spectrometry (Fig. S29).

**Figure 4:**
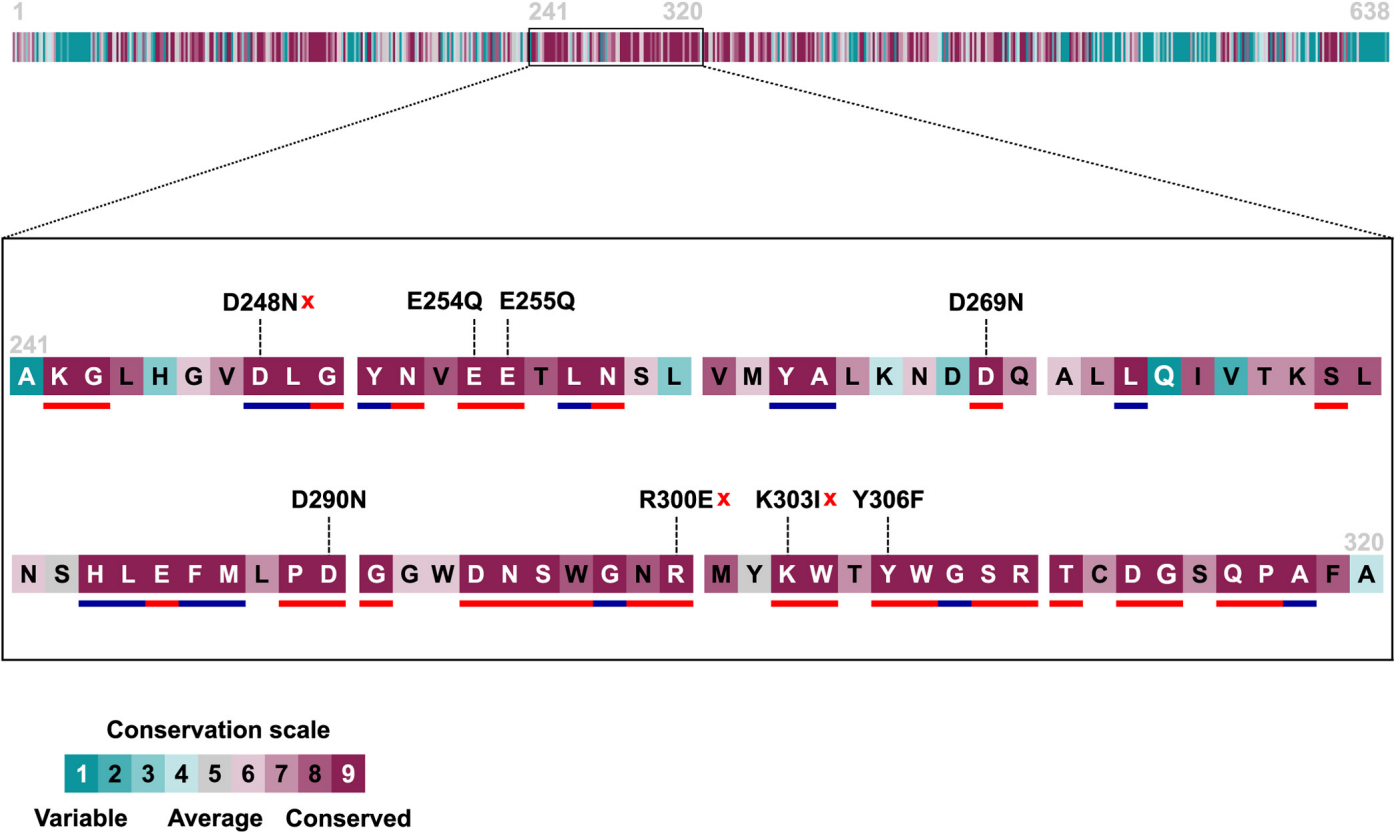
**Conserved residues of P29_PDnc sequence WP_038530528.1.** Conservation was determined using the ConSurf Server with the multiple sequence alignment of 25 phmmer hits *versus* UniProt reference proteomes given in Figure 6. This graphic was adapted from the graphical output of the ConSurf Server. The amino acids with a high conservation score (*purple*) and functional prediction (*red lines* under the main sequence) were chosen for the mutations to investigate for functionality of the P29_PDnc. Mutations, which lead to inactivation of the P29_PDnc, are marked with a *red cross*. *Blue underlined* residues are predicted as structural residue.

**Figure 5:**
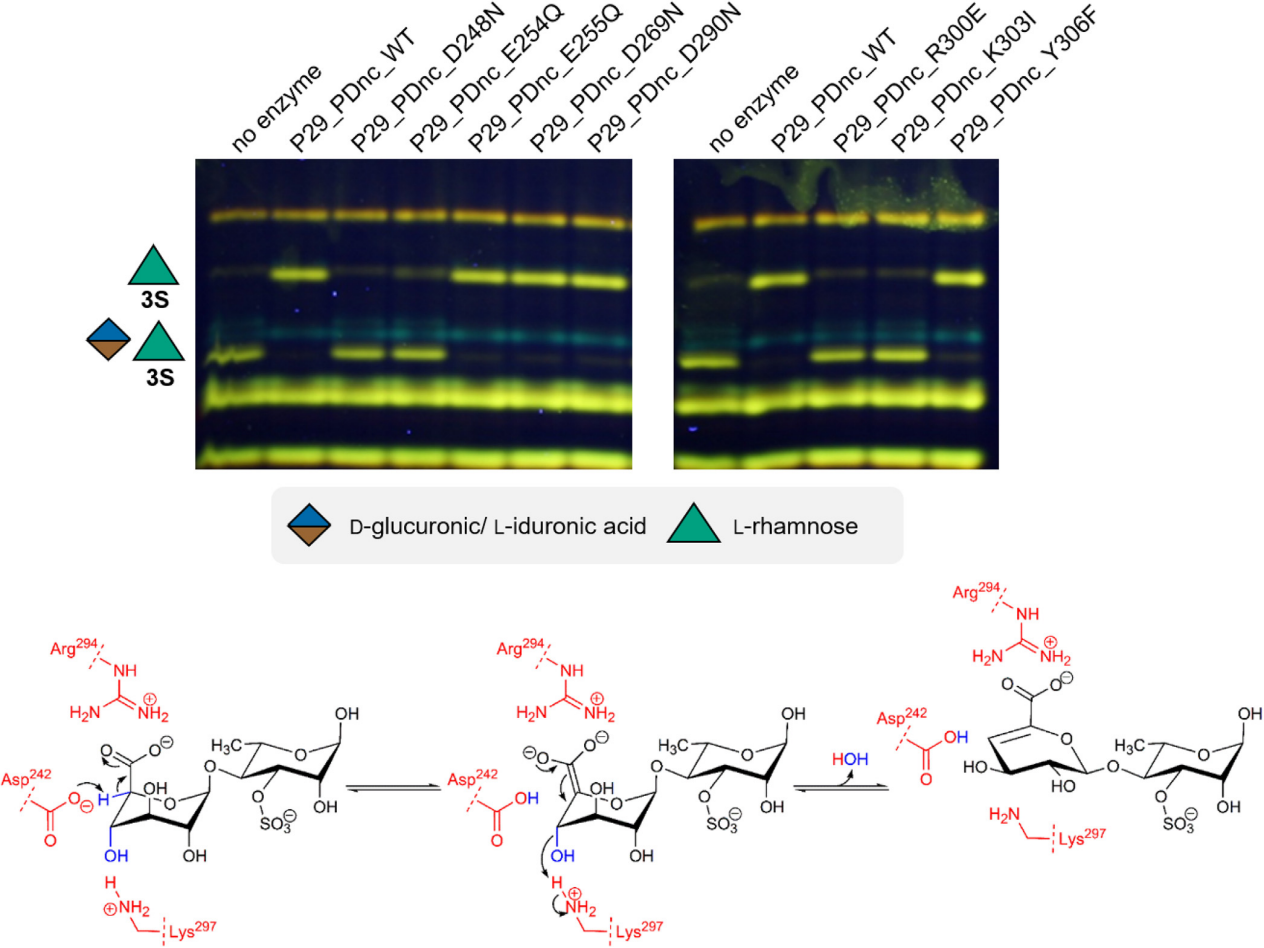
**Proposed preliminary reaction mechanism for the polysaccharide dehydratase P29_PDnc.** The variants of P29_PDnc were incubated with the disaccharide GlcA/IdoA-Rha3S produced from Δ-Rha3S-GlcA/IdoA-Rha3S with enzymes from the alternative pathway leading to the formation of the free α-keto acid and Rha3S. For the negative control, no P29_PDnc-single point mutation variant was added to the enzyme reaction with the enzymes from the alternative pathway. The P33_GH105 was not heat-inactivated as the produced rhamnose-3-sulfate can be distinguished much easier from GlcA/IdoA-Rha3S. A number in combination with an 'S' attached to a sugar represents the position of sulfate groups. “Unsaturated uronic acid” represents 4-deoxy-α-l-threo-hex-4-enopyranuronic acid. The mechanism shows the proposed reaction for the disaccharide β-d-glucuronic acid 1,4-linked to α-l-rhamnose-3-sulfate. For simplification, only the preliminary functional amino acid side chains are shown (*red*). The C5 proton (*blue*) is abstracted by Asp242, while Arg294 stabilizes the oxyanion intermediate. With support of Lys297, which protonates the glycosidic oxygen atom, water is eliminated under formation of the characteristic 5-dehydro-4-deoxy-d-glucuronate.

However, the true mechanism was elusive, as an oligosaccharide dehydratase of this type had never been reported before. When comparing the two most closely related mechanisms—that of PLs and of monosaccharide dehydratases—there are less similarities of P29_PDnc with the sugar dehydratases, as they all dehydrate side chains of monosugars, leading to an elimination of water from a side chain hydroxyl group, forming a desoxy sugar. The monosugar dehydratases convert their substrate *via* an oxidation mechanism (26, 27). Furthermore, P29_PDnc seems to be cofactor-independent as the reaction worked without addition of any supplements. The activity of P29_PDnc leads to a dehydration of a ring hydroxyl group resulting in the formation of an unsaturated hexenuronic acid residue. It seems to require a C5 carboxyl group like common PLs. Thus, the mechanism follows most probably the general PL mechanism, but eliminating water instead of a sugar residue. To reveal residues that are presumably involved in this catalysis, alignment studies were performed. Variants of P29_PDnc were produced with mutation of single functional amino acid residues (Fig. 4) to residues with similar structure, but different chemical properties, to ensure that these mutations lead to a loss of activity, because the residue is important for the catalysis and that not a structural change of the active site leads to the loss of activity. The P29_PDnc variants D248N, E254Q, E255Q, D269N, D290N, R300E, K303I, and Y306F were generated (Fig. 4). Interestingly, the residue Y306F showed still activity, after mutating it to phenylalanine as there was full conversion as shown in the FACE analysis (Fig. 5). Tyrosine is the catalytic residue in ulvan lyases as well as in many dehydratases (15, 26, 27), which led to the assumption that this is also the case for the novel ulvan PUL encoded dehydratase described here. As the mutation did not inactivate this variant, a catalytic participation of Y306 is rather unlikely. Three mutations led to a loss of activity indicated by the lack of conversion observed by the FACE analysis: D248N, R230E and K303I (Fig. 5). The arginine (R230) is presumably involved in the stabilization of the C5 carboxyl group of the substrate as it was reported to be the case in some PLs (13). The aspartate (D248N) most probably serves as the catalytic base abstracting the ring proton, which in turn initializes the formation of the enolate intermediate like it is the case in the lyase mechanism (13). The lysine (K303I) presumably provides a proton for the leaving group water and takes the role of the catalytic acid (13). These results led to the proposed preliminary mechanism shown in Figure 5.

## Discussion

In this study, we were able to complement the complex ulvan degradation pathway previously described by Reisky *et al.* (10) by elucidating an alternative enzyme cascade, which is able to fully degrade uronic-acid-containing oligosaccharides resulting from an incomplete degradation by ulvan lyases. Biochemical characterization of each step of the cascade with purified enzymes and structural determination of the produced intermediates enabled us to discover a new branch of the complex ulvan degradation pathway in *F. agariphila* (Fig. 2, see Fig. S1 for a comparison). In addition to the previously described ulvan-degrading enzymes (P10_PL40, P17_GH2, P18_S1_7, P20_GH78, P24_GH3, P27_GH43, P30_PL28, P31_GH39, P32_S1_8, P33_GH105, P36_S1_25), we were able to elucidate the function of three further enzymes in the ulvan utilization comprising two glucoside hydrolases (P34_GH3, P36_GH78) and an oligosaccharide dehydratase (P29_PDnc). P36_GH78 could also be confirmed to be the first multimodular enzyme participating in ulvan degradation as enzyme P36 combines a sulfatase domain with a GH78 domain. These two domains were analyzed separately. Furthermore, a new substrate specificity in this pathway could be observed for the previously described sulfatase of family S1_25 (P36_S1_25).

**Figure 6:**
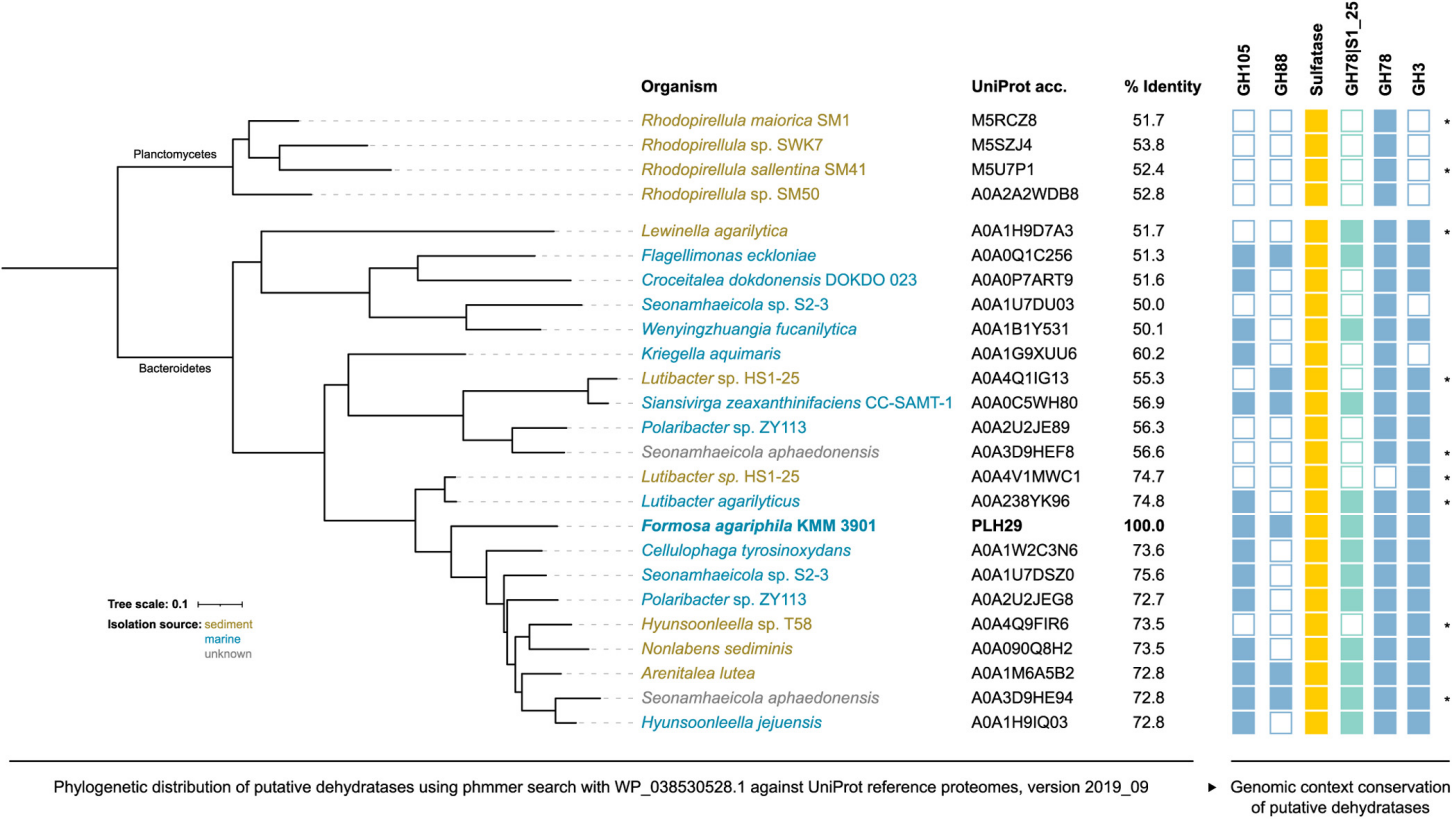
**Phylogenetic distribution of putative dehydratases in different organisms.** Phylogenetic analysis was done using the phmmer web server search with WP_038530528.1 against UniProt reference proteomes, version 2019_09. Only sequences with a percentage identity of at least 50% are shown. The *right panel* shows the presence or absence of alternative pathway encoded CAZymes and sulfatases of the respective dehydratases. Gene cluster highlighted with an *asterisk* (∗) might be incomplete due to limited contig length.

We found that the here described new alternative ulvan specific degradation pathway is conserved in other marine Bacteroidetes (Fig. 1), whereas sediment-associated Planctomycetes encode a more distant dehydratase homolog without a GH105, GH88, and GH3 context (Fig. 6).

This elucidation of an alternative degradation pathway illustrates the complexity of the biological systems for marine ulvan degradation. It indicates the necessity of backup mechanisms for metabolic processes in order to get access and compete for the diversity of complex marine carbon sources in nature. Several small degradation cascades complement each other to break substrate compounds down to the monomeric level for the use of the structurally diverse polysaccharide ulvan. The here described ulvan specific subpathway in general and the newly described dehydratase activity in particular enable a more efficient ulvan utilization. The higher the ulvan concentration is, the more the ulvan lyases are inhibited by their own products. This is prevented by the proposed alternative pathway, which is able to take over the function of the inhibited lyase cascade to ensure a more efficient utilization of ulvan sugars as energy and carbon sources.

The findings of this study expand our insights into the metabolic processes of the degradation of a complex marine polysaccharide and thus help to elucidate specific molecular mechanisms of the ocean’s carbon cycle. The characterizations of ulvan-active enzymes and the clarification of their substrate scopes allow using these enzymes for the production of ulvan-derived chemical products from currently rarely used green algal biomass.

## Experimental procedures

### Comparative genomics

Bacterial genomes were chosen according to (10) and downloaded from NCBI-GenBank. Ulvan PUL encoded carbohydrate-active enzymes were identified using the dbCAN meta server (http://bcb.unl.edu/dbCAN2) integrated tools HMMER, DIAMOND, and Hotpep (tool versions and databases as of 09/28/19) and assigned to CAZyme families if the carbohydrate-active enzyme was found by at least two tools (28). Putative dehydratases were predicted using the PL37 family. Sulfatases were annotated using HMMER v3.2.1 (29) against the Pfam profile PF00884. Pfam hits were further filtered with the dbCAN hmmer-scan-parser script and assigned to a sulfatase family using Protein-Protein BLAST v2.11.0+ (30) against the Sulfatlas database v1.1 (31) with an expect value threshold of 1E-5. Ulvan PULs were predicted by searching annotations of ulvan lyases and the surrounding genes using an up- and downstream distance to any CAZyme (excluding glycosyl transferases) or sulfatase of up to seven genes. Circos was used to visualize the results (32).

In order to determine a broader phylogenetic distribution and therefore substrate specificity, the P29_PDnc sequence WP_038530528.1 was searched against the uniprotrefprot database v2019_09 using phmmer v3.3.2 with the following settings “-E 1–domE 1–incE 0.01–incdomE 0.03–mx BLOSUM62–pextend 0.4–popen 0.02”, including all taxa (33). Hits with a percentage identity of at least 50% were aligned using CLUSTALW (34) with default settings. Maximum-likelihood phylogeny was estimated using PhyML webserver (default settings) (35, 36). The resulting tree was visualized with iTOL (37). Genomes of putative dehydratase encoding organisms were downloaded and analyzed as described above. Functionally and structurally important residues of WP_038530528.1 were analyzed and visualized using ConSurf web server (38, 39).

### Gene cloning

Expression constructs were prepared using the FastCloning strategy (40) with genomic DNA from *F. agariphila* KMM 3901T (collection number DSM15362 at DSMZ) as template for the amplification of the inserts. Generally, the pET28 constructs were prepared as described previously (10) with the gene primers shown in Table S4. To clone the gene for the formylglycine-generating enzyme (FGE) from *F. agariphila*, the vector backbone was amplified with the primers 5′-AATA GCGC CGTC GACC ATCA TCAT CATC ATCAT-3′ and 5′-CATG GTTA ATTC CTCC TGTT AGCC CAAA AA-3′ from pBAD/myc-his A (Addgene). The gene of the sulfatase P36_S1_25 was ordered codon-optimized for *Escherichia coli* from Genscript and subcloned into pET28 with *Nhe*I and *Xho*I. The optimized nucleotide sequence is provided in the Supporting information.

The gene of the glycoside hydrolase P36_GH78 was a kind gift from Gurvan Michel (Station Biologique de Roscoff, Roscoff, France).

### Enzyme production

*E. coli* BL21(DE3) was transformed with pET28-based plasmids harboring the required genes. For the overexpression, 50 ml LB or TB medium with 100 μg ml^−1^ kanamycin was inoculated from an overnight culture in LB containing 50 μg ml^−1^ kanamycin. The culture was grown at 37 °C and 180 rpm until the OD600 nm reached 0.8. The expression was then induced by adding 0.5 or 1 mM isopropyl β-d-1-thiogalactopyranoside (IPTG), and the culture was cooled to 20 °C (or to 16 °C in case of the P29-variants), for 24 h. For the expression of sulfatase genes, the FGE from *F. agariphila* was coexpressed. LB medium with 100 μg ml^−1^ ampicillin and 50 μg ml^−1^ kanamycin was inoculated from an overnight culture in the same medium and incubated at 37 °C and 180 rpm until the OD600 nm reached 0.3 to 0.5. After the addition of 1.5 mM l-arabinose and incubation for 90 min at 37 °C, the culture was cooled to 18 °C for 2 h before 0.5 mM IPTG was added, and the culture was incubated overnight at 18 °C.

### SDS-PAGE

Samples from the cultivations equivalent to a volume of 7/OD600 nm in ml were taken before harvest and the cells were collected by centrifugation (13,000*g*, 4 °C, 2 min). Pellets were resuspended in 500 μl 50 mM HEPES with 100 mM NaCl (pH 7.4). After lysis with FastPrep cell disruptor (MP Biomedicals), whole cell protein (W) samples were obtained prior to removal of the cell debris by centrifugation (13,000*g*, 4 °C, 10 min). Samples of the soluble protein fraction (S) were taken from the respective supernatant. For the SDS-PAGE, 12.5% acrylamide gels were used containing 1% (v/v) 2,2,2-trichloroethanol for the visualization of proteins under UV light (41). Electrophoresis was carried out at 200 V and gels were placed on a UV transilluminator for 2 min to develop the fluorescence after which pictures were taken. Alternatively, proteins were stained with Coomassie Blue (PhastGel Blue R, Sigma Aldrich).

### Enzyme purification

The cell pellets of a 50 ml culture were thawed on ice and resuspended in 10 ml of ice-cold Tris-HCl buffer (50 mM, pH 7.4 + 300 mM NaCl +10 mM imidazole) (wash buffer). The cells were lysed by ultrasonication on ice (2 × 3 min, 50% power, 50% cycle time), and the cell debris was removed by centrifugation (15 min at 10,000*g*). Rotigarose-His/Ni beads (Carl Roth) incubated with the clarified lysate were used in gravity flow columns. After washing, the protein was eluted with Tris-HCl-buffer (50 mM, pH 7.4 + 100 mM NaCl +300 mM imidazole). Fractions containing the protein of interest were pooled and desalted using PD-10 columns (GE Healthcare) equilibrated with 50 mM Tris-HCl (pH 7.4 + 10 mM NaCl). Alternatively, the same buffers with pH 8.0 instead of 7.4 could be used without any effect on the protocol. The desalted enzymes were aliquoted in tubes flash frozen in liquid nitrogen and stored at −20 °C. The protein concentration was determined with the Roti-Nanoquant kit with an albumin standard (0–100 μg/ml).

### Purification of ulvan

*Ulva* sp. was collected near Roscoff (France), Lubmin (Germany), or Helgoland (Germany) and dried. Alternatively, dried *Ulva* biomass from the Atlantic coast in Spain was purchased as organic sea lettuce (Kulau). Ulvan was extracted according to the literature (42). The dialysis step was exchanged by precipitation with acetone (80% (v/v) final concentration). After washing, acetone-precipitated ulvan was dissolved in deionized water and freeze-dried.

### Fluorophore-assisted carbohydrate electrophoresis

Fluorophore-assisted carbohydrate electrophoresis (FACE) was performed with 2-aminoacridone (AMAC) as fluorophore as shown previously (43).

### Carbohydrate polyacrylamide gel electrophoresis

For carbohydrate polyacrylamide gel electrophoresis (C-PAGE), samples were mixed with an equal volume of FACE loading buffer (42). Gels and running conditions were identical to FACE. Carbohydrates were visualized by staining with Stains-All solution (0.25 g l^−1^ in 1.7 mM Tris-HCl pH 7.5 + 25% (v/v) isopropanol). The gels were destained with 35% (v/v) isopropanol in deionized water.

### Purification of oligomers and structure determination

Ulvan (300 mg) was digested with purified enzymes (100 μg/ml) in 35 mM Tris-HCl buffer (pH 8.0 + 50 mM NaCl) at room temperature overnight. Oligomers were separated on two XK 26/100 (GE Healthcare) in row filled with Bio-Gel P-2 (Rio-Rad) using 50 mM (NH_4_)_2_CO_3_ as mobile phase at a flow rate of 1 ml min^−1^. After lyophilization of the fractions containing the products, oligomers were dissolved in D_2_O and lyophilized two times before NMR spectra were recorded on a Bruker Avance III HD 600 (600 MHz) spectrometer (Bruker) in D_2_O solutions. The oligomers—with the confirmed NMR structures—served as standards for activity screening of PUL H enzymes and fractioning after size-exclusion chromatography. The degradation was performed stepwise. The structures were independently elucidated based on 1D and 2D (COSY, HSQC, HMBC, TOCSY) methods, and the assigned ^1^H and ^13^C-NMR signals were then compared with literature data, showing excellent consistency (18, 22, 44). For samples containing uronic acid structures, it was required to neutralize the otherwise acidic NMR samples with Na_2_HPO_4_ to pH 7 to 8 (pH-electrode calibrated to H^+^) in order to achieve fully resolved signals for the carboxylic acid and neighboring positions (^13^C). HPLC−ELS-MS analysis was performed by injection of ∼0.1% solutions (1–5 μl) on a Nexera UHPLC system from Shimadzu (equipped with two binary LC-30AD pumps plus degassers, a CTO-20 column oven) and an LCMS-2200 EV MS-detector and an additional ELS-detector (JASCO ELS-2041). Analysis was performed with mobile phase A = H_2_O (0.1% HCOOH) and mobile phase B = CH_3_CN on a C_18_ column (XSelect CSH XP C18 2.5 μm 3 × 50 mm) at 40 °C. Flow rate was 1.3 ml min^−1^ (0–3 min) with 5% B from 0 to 0.15 min, 5% to 98% B from 0.15 to 2.2 min and 98% to 5% B from 2.2 to 2.5 min.

### Enzyme assays

Generally, reactions were performed in 35 mM Tris-buffer (pH 8.0 + 50 mM NaCl) ensuring addition of sufficient amounts of the respective enzyme. The ulvan degradation products and the conversion of purified oligomers were analyzed by FACE. Untreated ulvan was generally at a concentration of 1 g l^−1^ while purified oligomers were used at 0.25 mg ml^−1^. Incubation was performed overnight at room temperature.

For ulvan lyase activity detection, the respective purified enzymes (15 μg/ml, see also Table S5) were added to an ulvan solution of 1 g l^−1^ in Tris-buffer (35 mM, pH 8.0, 50 mM NaCl), and the increase of absorbance at 235 nm was measured over time. A sample of the breakdown products was analyzed with the MBTH-assay (45) adapted for reduced volumina (200 μl) and C-PAGE. For the detection of 5-dehydro-4-deoxy-d-glucuronate after reaction with P29_PDnc variants with N-terminal or C-terminal His-tag and/or P33_GH105, the thiobarbituric acid assay (23) adapted for reduced volumina has been used. In total, 37.5 μl of the reaction mixture was mixed with an equal volume of 2% (w/v) sodium acetate in 0.5 N HCl, followed by the addition of 150 μl 0.3% (w/v) thiobarbituric acid in distilled H_2_O.

## Data availability

All data of this study are contained within the article.

## Supporting information

This article contains supporting information (10, 18).

## Conflict of interest

The authors declare no conflict of interest.
