# Supplementary material for: A new carbohydrate-active oligosaccharide dehydratase is involved in the degradation of ulvan
Source: J Biol Chem. 2021 Sep 20;297(4):101210. doi: 10.1016/j.jbc.2021.101210 (PMC8511951; doi:10.1016/j.jbc.2021.101210)
Supplement: Supplemental Figs. S1–S29 and Tables S1–S6 [file mmc1.pdf]

Supporting Information

A new carbohydrate-active oligosaccharide dehydratase is involved in the degradation of ulvan

Marcus Bäumgen[1,‡], Theresa Dutschei[1,‡], Daniel Bartosik[2], Christoph Suster[3], Lukas Reisky[1], Nadine Gerlach[4,5], Christian Stanetty[3], Marko D. Mihovilovic[3], Thomas Schweder[2], Jan-Hendrik Hehemann[4,5], and Uwe T. Bornscheuer[1,*]

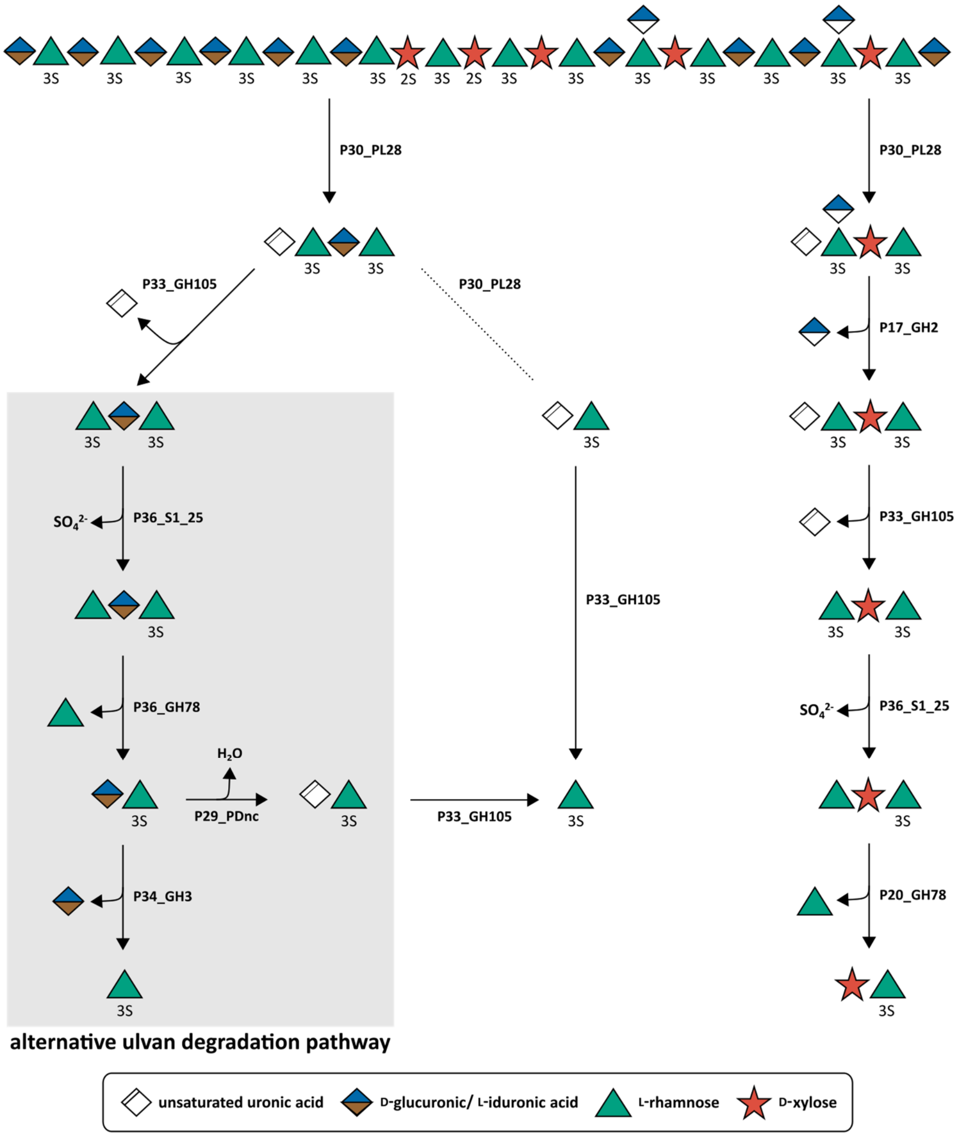

Figure S1. Overview of the degradation of uronic acid containing ulvan oligosaccharides from *Formosa agariphila* KM3901T by PUL H enzymes. The left side summarizes the novel found activities which were presented in this study, which complements the pathway described by Reisky *et al.* 2019 (10). This alternative pathway fills the gap in the degradation of the ulvan oligosaccharides as the previous pathway lacked the degradation of small uronic acid containing oligosaccharide as the ulvan lyase P30_PL28 cannot be active due to product inhibition (dotted line) (18).

Table S1a. NMR shifts of GlcA-trimer contained in a mixture of Rha3S-GlcA-Rha3S and Rha3S-IdoA-Rha3S (ratio ~7:3) originating from the digestion of starting tetramer (Δ-Rha3S-GlcA-Rha3S) with Enzyme P33_GH105. The chemical shifts are compared to the starting material (10,18). Indicative shifts of chemical shifts that support the claimed structure are marked in red. NMR spectra are shown in Figs. S2 to S4. ESI-MS measurements support the structure (Fig. S6 and Fig. S7). The shifts of the IdoA-component are shown in Table S1b.

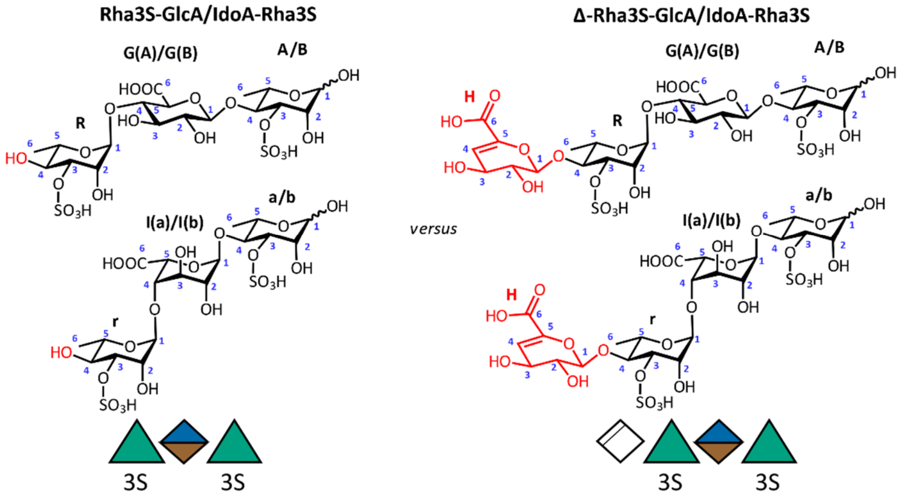

		^{1}H-Shifts (ppm)			^{13}C Shifts (ppm)		
		**Rha3S-GlcA Rha3S**	**Ref** Δ-Rha3S-GlcA-Rha3S **(10)**	**Delta (A1=A1)**	**Rha3S-GlcA Rha3S**	**Ref** Δ-Rha3S-GlcA-Rha3S **(10)**	**Delta (A1=A1)**
**A** α-Rhamnose	**1**	**5.08**	**5.08**	**0.00**	**96.1**	**94.62**	**0.00**
	**2**	4.23	4.21	0.02	71.98	70.50	0.00
	**3**	4.61	4.61	0.00	80.73	79.25	0.00
	**4**	3.76	3.77	-0.01	81.02	79.48	0.06
	**5**	3.98	3.98	0.00	70.3	68.82	0.00
	**6**	1.31	1.30	0.01	19.89	18.41	0.00
**B** β-Rhamnose	**1**	4.89	4.89	0.00	95.67	94.19	0.00
	**2**	4.23	4.23	0.00	72.41	70.93	0.00
	**3**	4.43	4.42	0.01	82.68	81.20	0.00
	**4**	3.69	3.68	0.01	80.58	79.03	0.07
	**5**	3.53	3.52	0.01	73.54	72.05	0.01
	**6**	1.32	1.31	0.01	19.89	18.41	0.00
**G(A)** Glucuronic acid (of α-anomer)	**1**	4.65	4.64	0.01	105.85	104.35	0.02
	**2**	3.33	3.31	0.02	76.44	74.84	0.12
	**3**	3.65	3.63	0.02	76.44	74.96	0.00
	**4**	3.58	3.56	0.02	81.51	80.11	-0.08
	**5**	3.85	3.84	0.01	77.8	76.32	0.00
	**6**				177.13	175.57	0.08
**G(B)** Glucuronic acid (of β-anomer)	**1**	4.65	4.64	0.01	105.87	104.32	0.07
	**2**	3.32	3.30	0.02	76.44	74.88	0.08
	**3**	3.65	3.63	0.02	76.44	74.96	0.00
	**4**	3.58	3.55	0.03	81.51	80.11	-0.08
	**5**	3.85	3.84	0.01	77.8	76.32	0.00
	**6**				177.13	175.57	0.08
**R** Rhamnoside	**1**	4.73	4.72	0.01	102.97	101.34	0.15
	**2**	4.23	4.19	0.04	71.42	70.1	-0.16
	**3**	4.42	4.58	-0.16	81.17	79.93	-0.24
	**4**	3.53	3.77	-0.24	72.52	77.67	-6.63
	**5**	4.11	4.12	-0.01	71.63	68.6	1.55
	**6**	1.25	1.11	0.14	19.23	17.63	0.12

Table S1b. NMR shifts of IdoA-trimer contained in a mixture of Rha3S-GlcA-Rha3S and Rha3S-IdoA-Rha3S (ratio ~7:3) originating from the digestion of starting tetramer (Δ-Rha3S-IdoA-Rha3S) with Enzyme P33_GH105. The chemical shifts are compared to the starting material[2]. Indicative shifts of chemical shifts that support the claimed structure are marked in red. NMR spectra are shown in Figs. S2 to S5. ESI-MS measurements support the structure shown in Fig. S6 and S7). The shifts of the GlcA-component are shown in Table S1a. Due to the low concentration of the IdoA component, some signals were not assignable with certainty, and are therefore omitted in the Table.

		^{1}H-Shifts (ppm)			^{13}C Shifts (ppm)		
		**Rha3S-IdoA Rha3S**	**Ref** Δ-Rha3S-IdoA-Rha3S **(10)**	**Delta (A1=A1)**	**Rha3S-IdoA Rha3S**	**Ref** Δ-Rha3S-IdoA-Rha3S **(10)**	**Delta (A1=A1)**
**a** α-Rhamnose	**1**	**5.08**	**5.08**	**0.00**	**96.34**	**94.86**	**0.00**
	**2**	4.23	4.22	0.01	72.09	70.61	0.00
	**3**	4.61	4.60	0.01	81.36	79.26	0.62
	**4**	3.76	3.76	0.00	79.07	77.51	0.08
	**5**	3.98	3.96	0.02	69.83	68.34	0.01
	**6**	1.2	1.18	0.02	19.71	18.23	0.00
**b** β-Rhamnose	**1**	4.89	4.88	0.01	95.82	94.34	0.00
	**2**	4.23	4.22	0.01	72.46	70.97	0.01
	**3**	4.43	4.41	0.02	83.22	81.74	0.00
	**4**	3.69	3.68	0.01	78.75	77.18	0.09
	**5**	3.53	3.51	0.02	73.16	71.68	0.00
	**6**	1.22	1.20	0.02	19.71	18.23	0.00
**I(A)** Iduronic acid (of α-anomer)	**1**	5.05	5.01	0.04	105.74	104.19	0.07
	**2**	3.63	3.59	0.04	73.29	71.79	0.02
	**3**	3.81	3.76	0.05	74.16	72.58	0.10
	**4**	3.98	3.96	0.02	82.12	80.61	0.03
	**5**	*	4.46		73.22	*	
	**6**				176.06	174.57	0.01
**I(B)** Iduronic acid (of β-anomer)	**1**	5.05	5.01	0.04	105.71	104.16	0.07
	**2**	3.63	3.59	0.04	73.29	71.72	0.09
	**3**	3.81	3.76	0.05	74.08	72.50	0.10
	**4**	3.98	3.96	0.02	82.06	80.54	0.04
	**5**	*	4.46		73.22	*	
	**6**				176.06	174.57	0.01
**r** Rhamnoside	**1**	4.86	4.82	0.04	104.03	102.39	0.16
	**2**	4.18	4.15	0.03	71.33	70.07	-0.22
	**3**	4.4	4.56	-0.16	81.17	79.88	-0.19
	**4**	3.53	3.76	-0.23	72.63	77.62	-6.47
	**5**	3.98	3.96	0.02	71.77	68.78	1.51
	**6**	1.27	1.12	0.15	19.29	17.71	0.1
*** no unambigous assignment possible**		*Referenced to HDO: 4.79 ppm*					

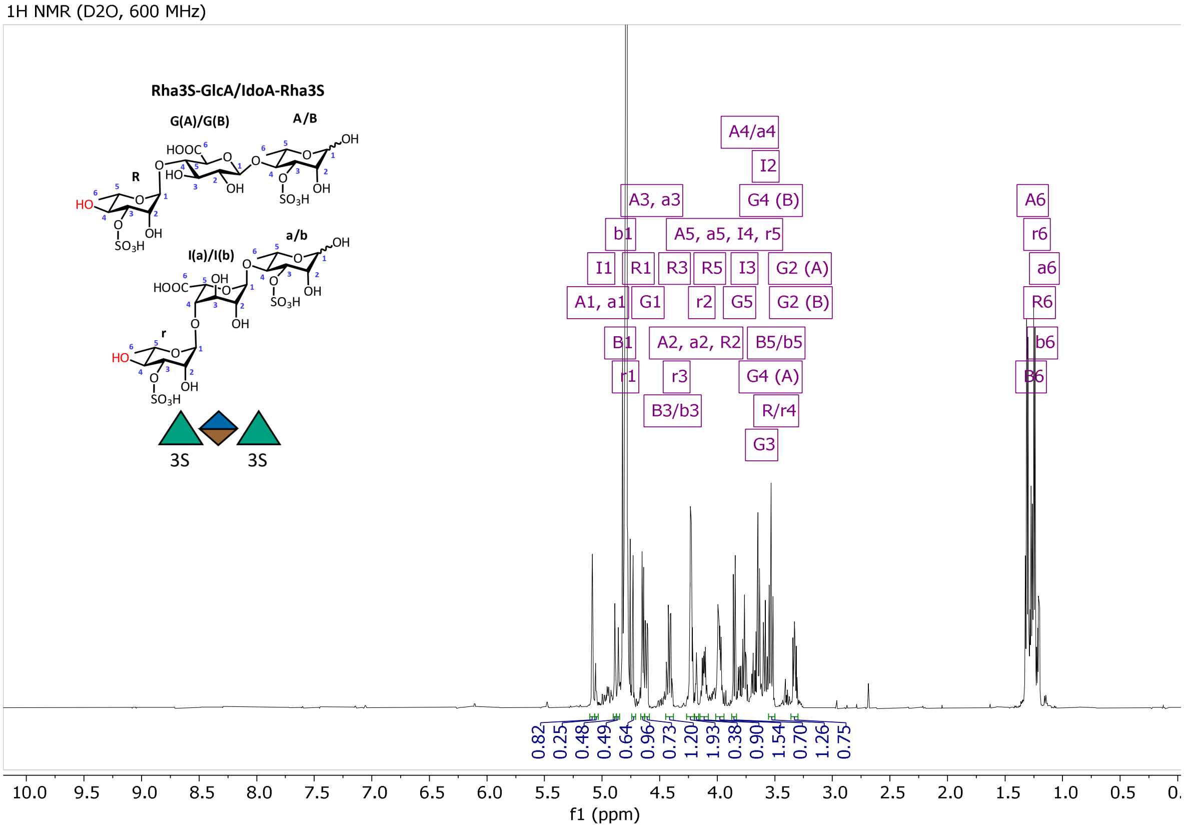

Figure S2. ¹H-NMR of the purified trimer mixture containing Rha3S-GlcA-Rha3S and Rha3S-IdoA-Rha3S (ratio ~7:3) – Full View

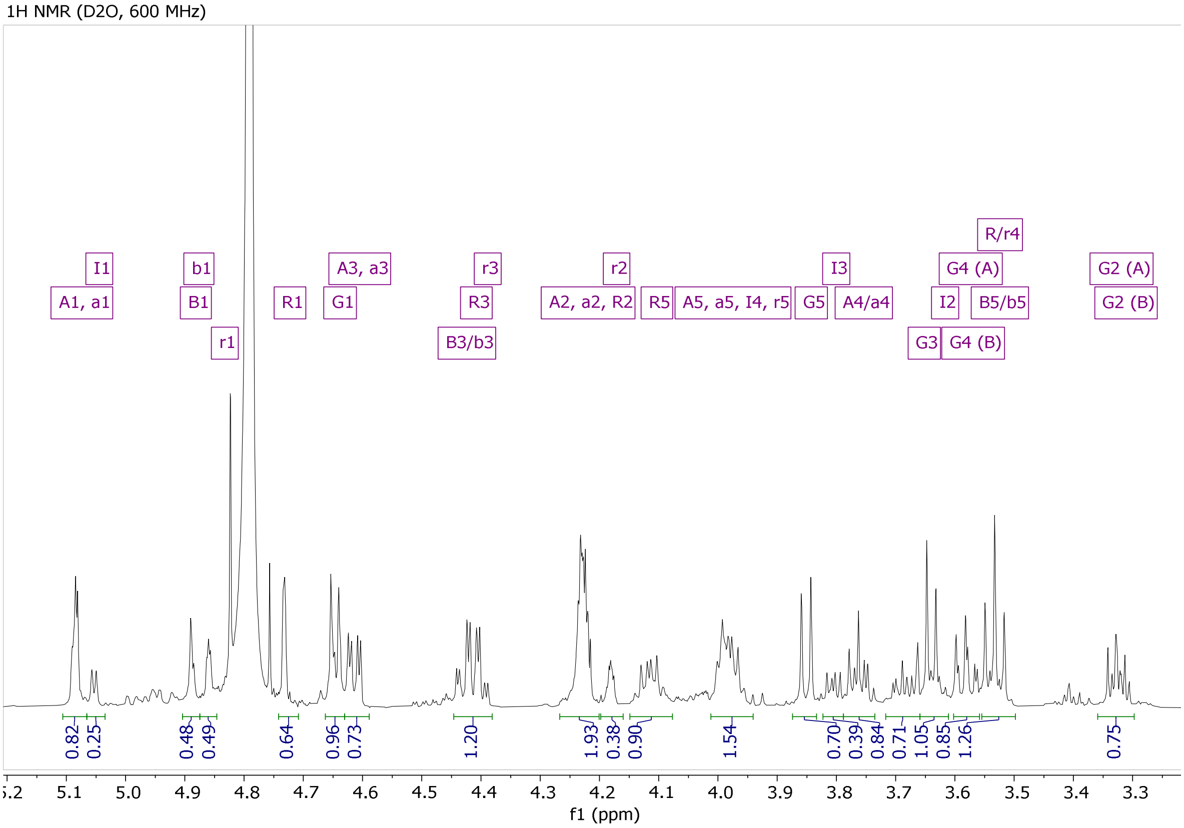

Figure S3. ¹H-NMR of the purified trimer mixture containing Rha3S-GlcA-Rha3S and Rha3S-IdoA-Rha3S (ratio ~7:3) – Zoom into the carbohydrate region

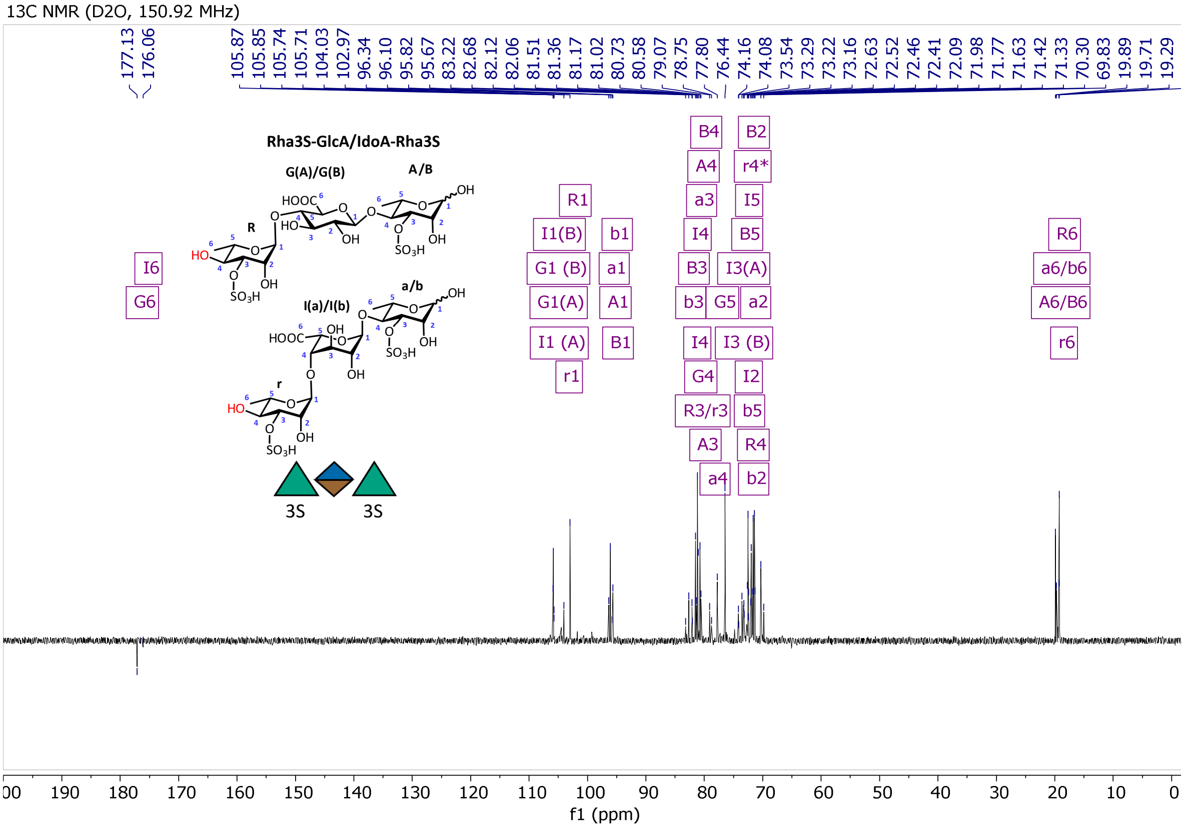

Figure S4. ^{13}C-NMR of the purified trimer mixture containing Rha3S-GlcA-Rha3S and Rha3S-IdoA-Rha3S (ratio ~7:3) – FullView

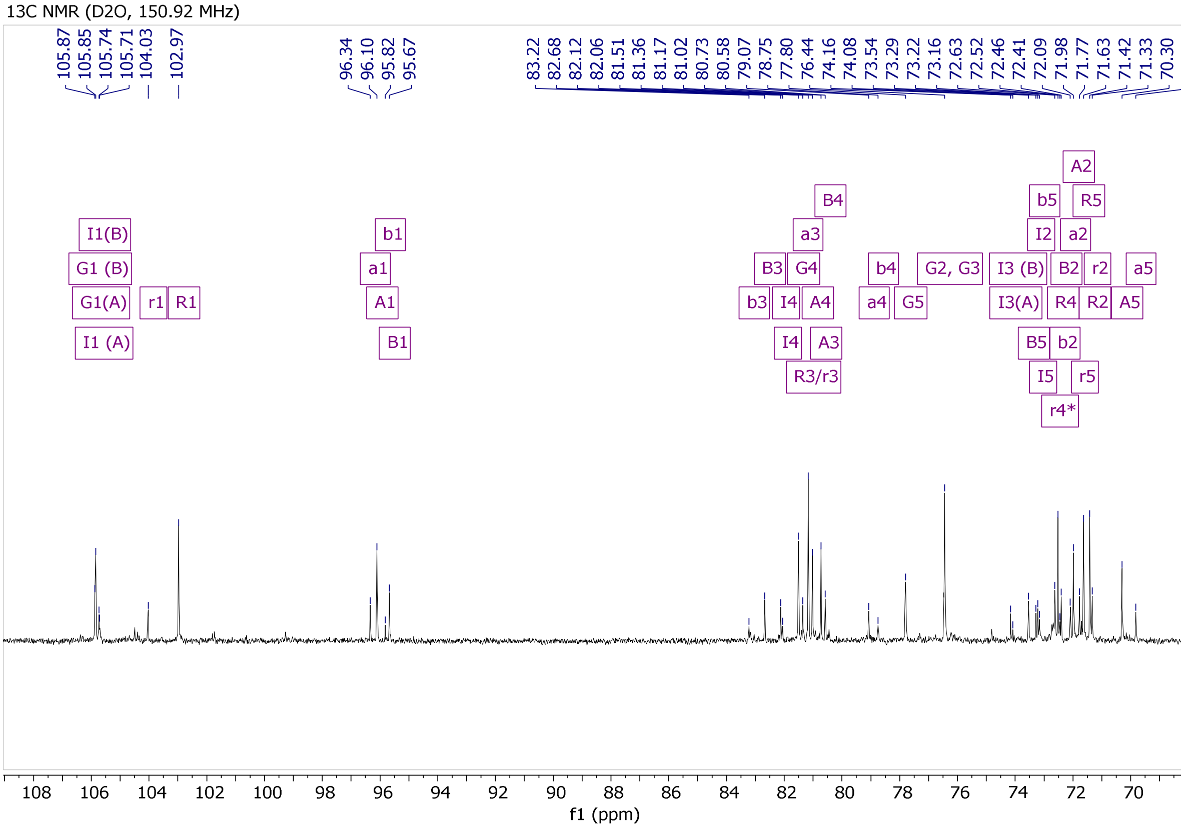

Figure S5. ^{13}C-NMR of the purified trimer mixture containing Rha3S-GlcA-Rha3S and Rha3S-IdoA-Rha3S (ratio ~7:3) – Zoom into the carbohydrate region

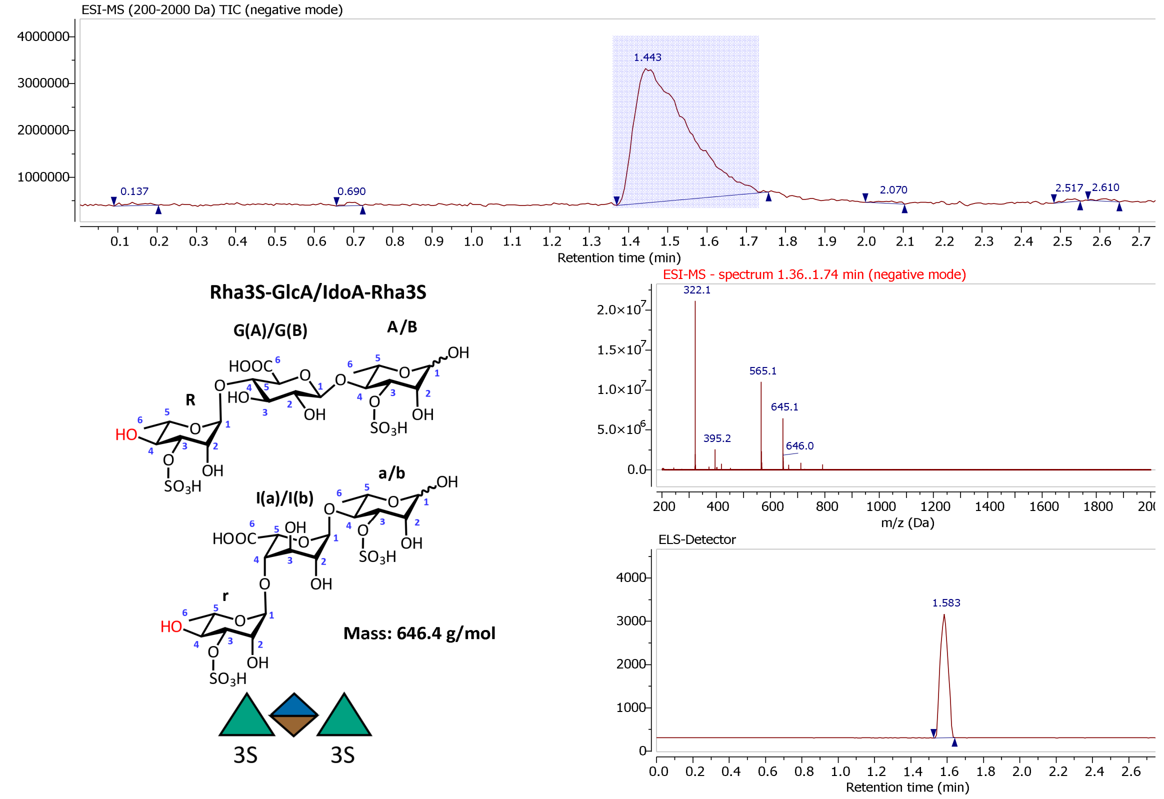

Figure S6. HPLC-MS (ESI-) measurement of the trimer mixture containing Rha3S-GlcA-Rha3S and Rha3S-IdoA-Rha3S (ratio ~7:3). Showing the [M-1] molecule peak of the compounds and also the [M-80] signal, which was shown to be only present in oligosaccharide structures containing SO_3 on a non-reducing sugar.

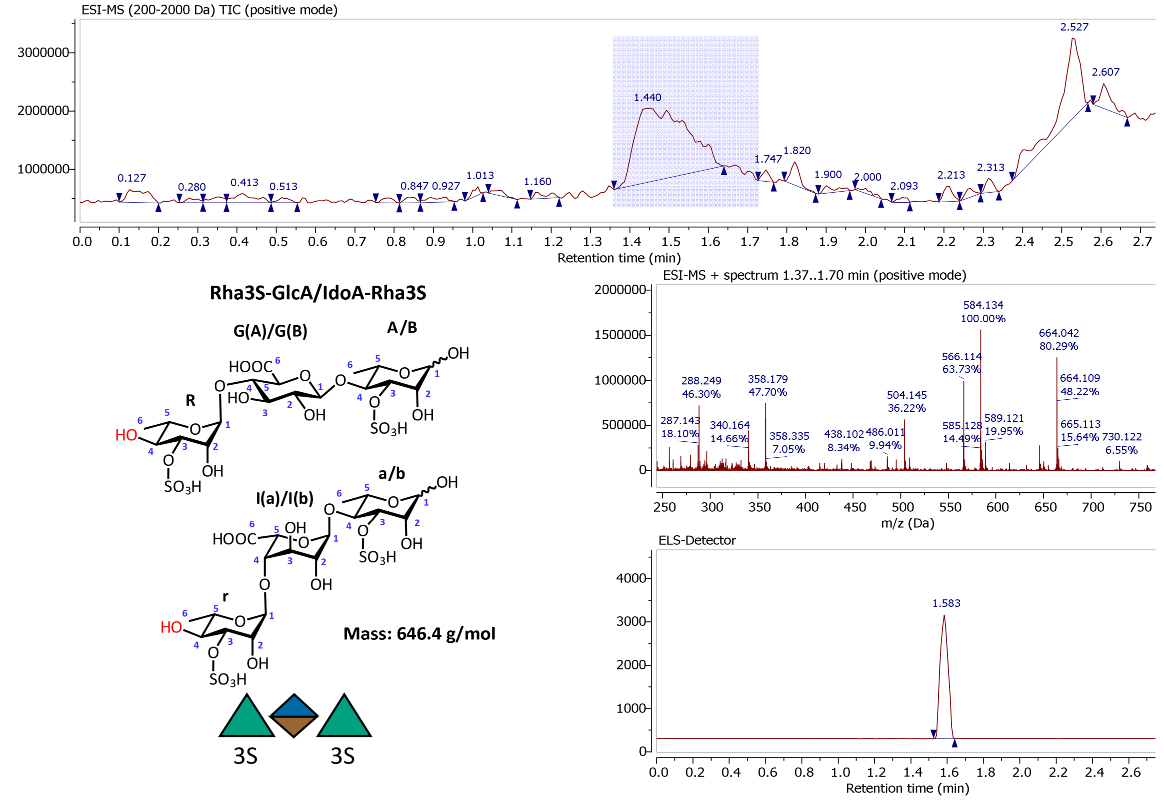

Figure S7. HPLC-MS (ESI+) measurement of the trimer mixture containing Rha3S-GlcA-Rha3S and Rha3S-IdoA-Rha3S (ratio ~7:3). Showing the [M+18] molecule peak of the compounds and also the [M-80+18] signal, which was shown to be only present in oligosaccharide structures containing SO_3 on a non-reducing sugar.

Table S2a. NMR shifts of GlcA-trimer contained in a mixture of Rha-GlcA-Rha3S and Rha-IdoA-Rha3S (ratio ~7:3) originating from the digestion of Rha3S-GlcA-Rha3S with Enzyme P36_S1_25. The chemical shifts are compared to the starting material. Indicative shifts of chemical shifts that support the claimed structure are marked in red. NMR spectra are shown in Figs. S8 to S11. ESI-MS measurements support the structure shown in (Figs. S12 and S13). The shifts of the IdoA-component are shown in Table S2b.

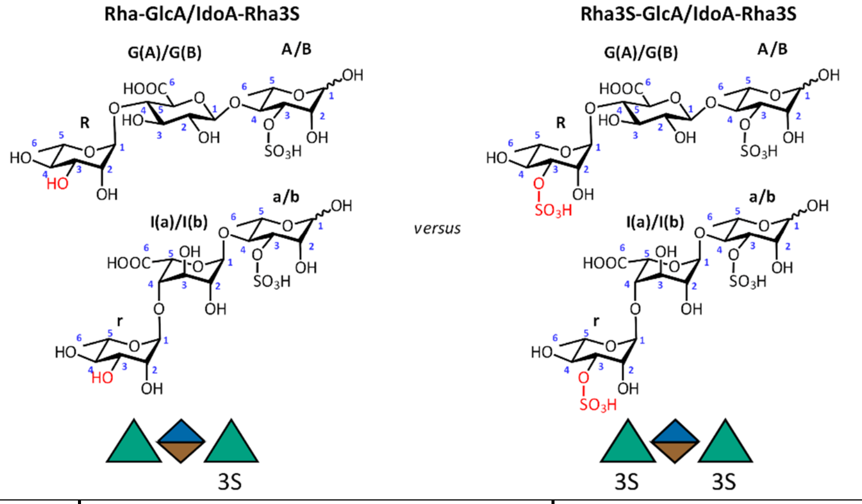

		^{1}H-Shifts (ppm)			^{13}C Shifts (ppm)		
		**Rha-GlcA Rha3S**	**Ref** Rha3S-GlcA-Rha3S	**Delta (A1=A1)**	**Rha-GlcA Rha3S**	**Ref** Rha3S-GlcA-Rha3S	**Delta (A1=A1)**
**A** α-Rhamnose	**1**	**5.08**	**5.08**	**0.00**	**96.12**	**96.1**	**0.00**
	**2**	4.22	4.23	-0.01	71.98	71.98	-0.02
	**3**	4.61	4.61	0.00	80.76	80.73	0.01
	**4**	3.75	3.76	-0.01	80.88	81.02	-0.16
	**5**	3.99	3.98	0.01	70.33	70.3	0.01
	**6**	1.31	1.31	0.00	19.91	19.89	0.00
**B** β-Rhamnose	**1**	4.89	4.89	0.00	95.67	95.67	-0.02
	**2**	4.23	4.23	0.00	72.42	72.41	-0.01
	**3**	4.43	4.43	0.00	82.7	82.68	0.00
	**4**	3.68	3.69	-0.01	80.46	80.58	-0.14
	**5**	3.52	3.53	-0.01	73.57	73.54	0.01
	**6**	1.32	1.32	0.00	19.91	19.89	0.00
**G(A)** Glucuronic acid (of α-anomer)	**1**	4.61	4.65	-0.04	105.83	105.85	-0.04
	**2**	3.33	3.33	0.00	76.48	76.44	0.02
	**3**	3.58	3.65	-0.07	76.7	76.44	0.24
	**4**	3.55	3.58	-0.03	81.86	81.51	0.33
	**5**	3.7	3.85	-0.15	78.85	77.8	1.03
	**6**				178.15	177.13	1.00
**G(B)** Glucuronic acid (of β-anomer)	**1**	4.61	4.65	-0.04	105.83	105.87	-0.06
	**2**	3.32	3.32	0.00	76.51	76.44	0.05
	**3**	3.58	3.65	-0.07	76.7	76.44	0.24
	**4**	3.53	3.58	-0.05	81.86	81.51	0.33
	**5**	3.7	3.85	-0.15	78.85	77.8	1.03
	**6**				178.15	177.13	1.00
**R** Rhamnoside	**1**	4.69	4.73	-0.04	103.36	102.97	0.37
	**2**	3.89	4.23	-0.34	72.97	71.42	1.53
	**3**	3.74	4.42	-0.68	72.65	81.17	-8.54
	**4**	3.38	3.53	-0.15	74.6	72.52	2.06
	**5**	3.99	4.11	-0.12	71.56	71.63	-0.09
	**6**	1.21	1.25	-0.04	19.1	19.23	-0.15
		Referenced to HDO: 4.79 ppm					

Table S2b. NMR shifts of IdoA-trimer contained in a mixture of Rha-GlcA-Rha3S and Rha-IdoA-Rha3S (ratio ~7:3) originating from the digestion of Rha3S-IdoA-Rha3S with enzyme P36_S1_25. The chemical shifts are compared to the starting material. Indicative shifts of chemical shifts that support the claimed structure are marked in red. NMR spectra are shown in Fig. S8 to S11. ESI-MS measurements support the structure shown in (Figs. S12 and S13). The shifts of the IdoA-component are shown in Table S2a. Due to the low concentration of the IdoA component, some signals were not assignable with certainty, and are therefore omitted in the Table.

		^{1}H-Shifts (ppm)			^{13}C Shifts (ppm)		
		**Rha-IdoA Rha3S**	**Ref** Rha3S-IdoA-Rha3S	**Delta (A1=A1)**	**Rha-IdoA Rha3S**	**Ref** Rha3S-IdoA-Rha3S	**Delta (A1=A1)**
**a** α-Rhamnose	**1**	5.08	5.08	0.00	96.38	96.34	0.00
	**2**	4.22	4.23	-0.01	72.1	72.09	-0.03
	**3**	4.61	4.61	0.00	81.47	81.36	0.07
	**4**	3.75	3.76	-0.01	*	79.07	
	**5**	3.99	3.98	0.01	*	69.83	
	**6**	*	1.2		19.7	19.71	
**b** β-Rhamnose	**1**	4.89	4.89	0.00	95.83	95.82	-0.03
	**2**	4.22	4.23	-0.01	72.6	72.46	0.10
	**3**	4.43	4.43	0.00	*	83.22	
	**4**	3.68	3.69	-0.01	78.62	78.75	-0.17
	**5**	3.52	3.53	-0.01	*	73.16	
	**6**	*	1.22		19.7	19.71	-0.05
**I(A)** Iduronic acid (of α-anomer)	**1**	5.02	5.05	-0.03	105.68	105.74	-0.10
	**2**	3.64	3.63	0.01	73.19	73.29	-0.14
	**3**	3.77	3.81	-0.04	*	74.16	
	**4**	3.99	3.98	0.01	*	82.12	
	**5**	*			*	73.22	
	**6**				177.25	176.06	1.15
**I(B)** Iduronic acid (of β-anomer)	**1**	5.02	5.05	-0.03	105.68	105.71	-0.07
	**2**	3.62	3.63	-0.01	73.19	73.29	-0.14
	**3**	3.77	3.81	-0.04	*	74.08	
	**4**	3.99	3.98	0.01	*	82.06	
	**5**	*	*		*	73.22	
	**6**				177.25	176.06	1.15
**r** Rhamnoside	**1**	*	4.86		104.47	104.03	0.40
	**2**	3.86	4.18	-0.32	72.94	71.33	1.57
	**3**	3.74	4.4	-0.66	72.62	81.17	-8.59
	**4**	3.38	3.53	-0.15	74.67	72.63	2.00
	**5**	3.89	3.98	-0.09	71.67	71.77	-0.14
	**6**	1.24	1.27	-0.03	19.16/19.33*	19.29	
***... assignment not possible**		*Referenced to HDO: 4.79 ppm*					

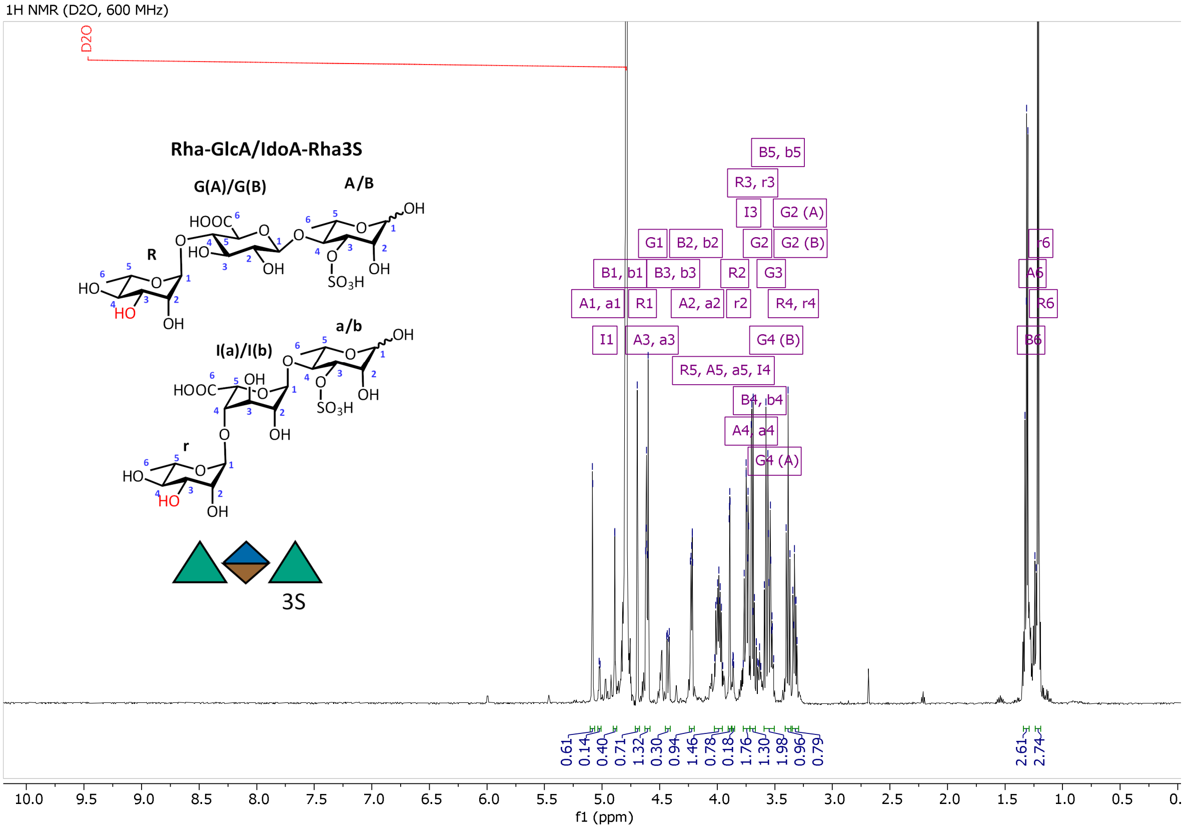

Figure S8. ^{1}H-NMR of the purified trimer mixture containing Rha-GlcA-Rha3S and Rha-IdoA-Rha3S (ratio ~7:3) – Full View

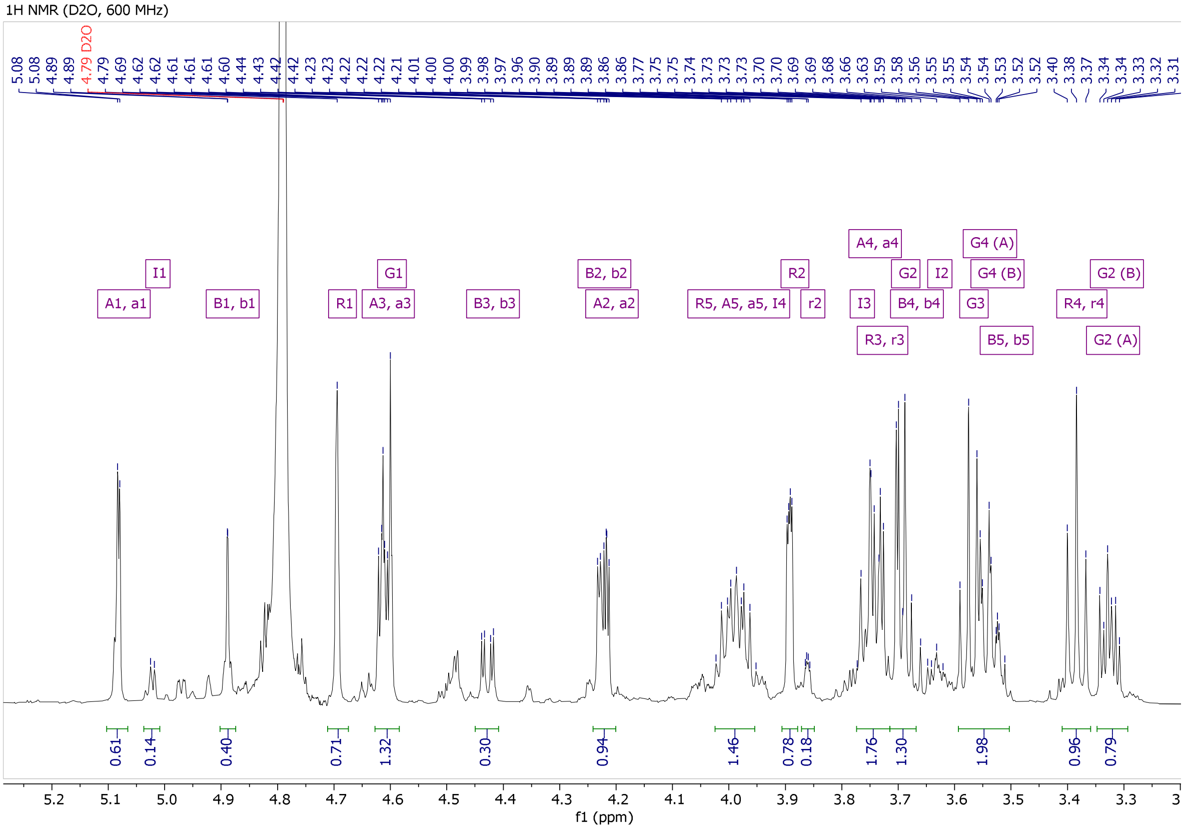

Figure S9. ^{1}H-NMR of the purified trimer mixture containing Rha-GlcA-Rha3S and Rha-IdoA-Rha3S (ratio ~7:3) – Zoom into the carbohydrate region

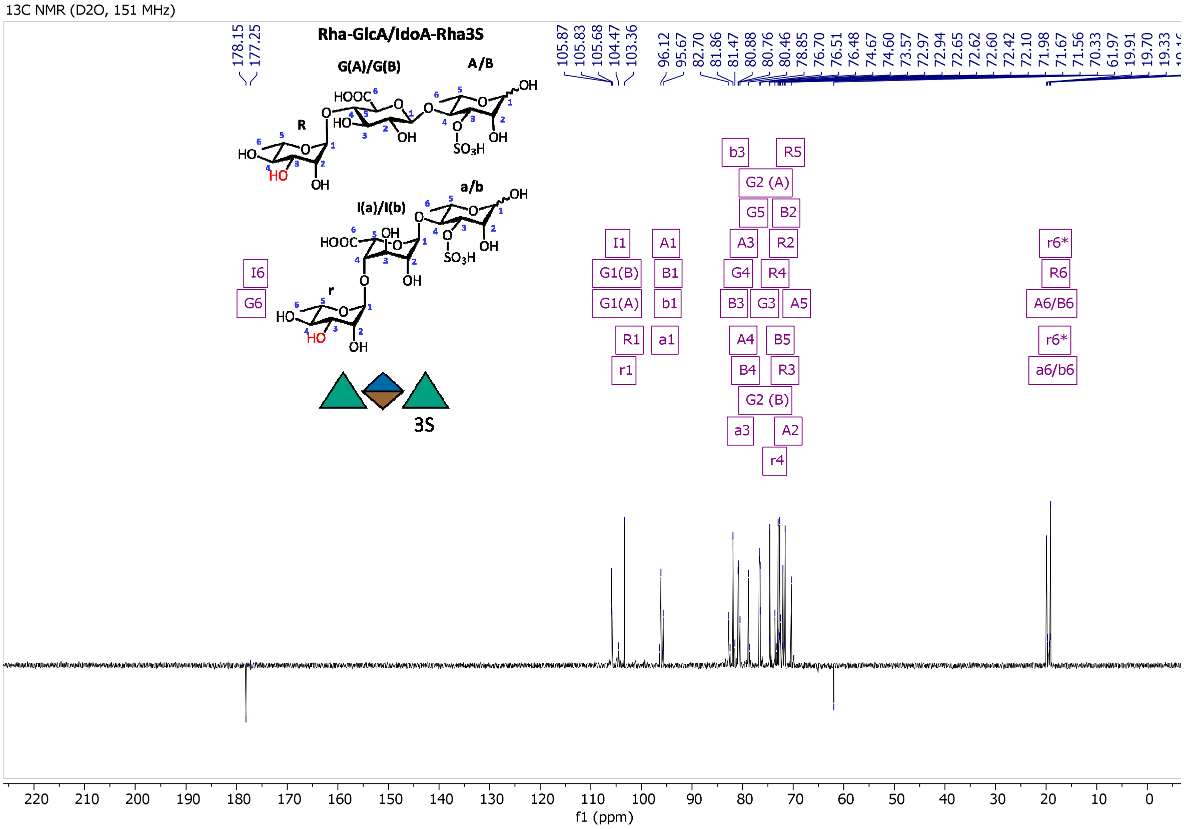

Figure S10. ^{13}C-NMR of the purified trimer mixture containing Rha3S-GlcA-Rha3S and Rha3S-IdoA-Rha3S (ratio ~7:3) – FullView

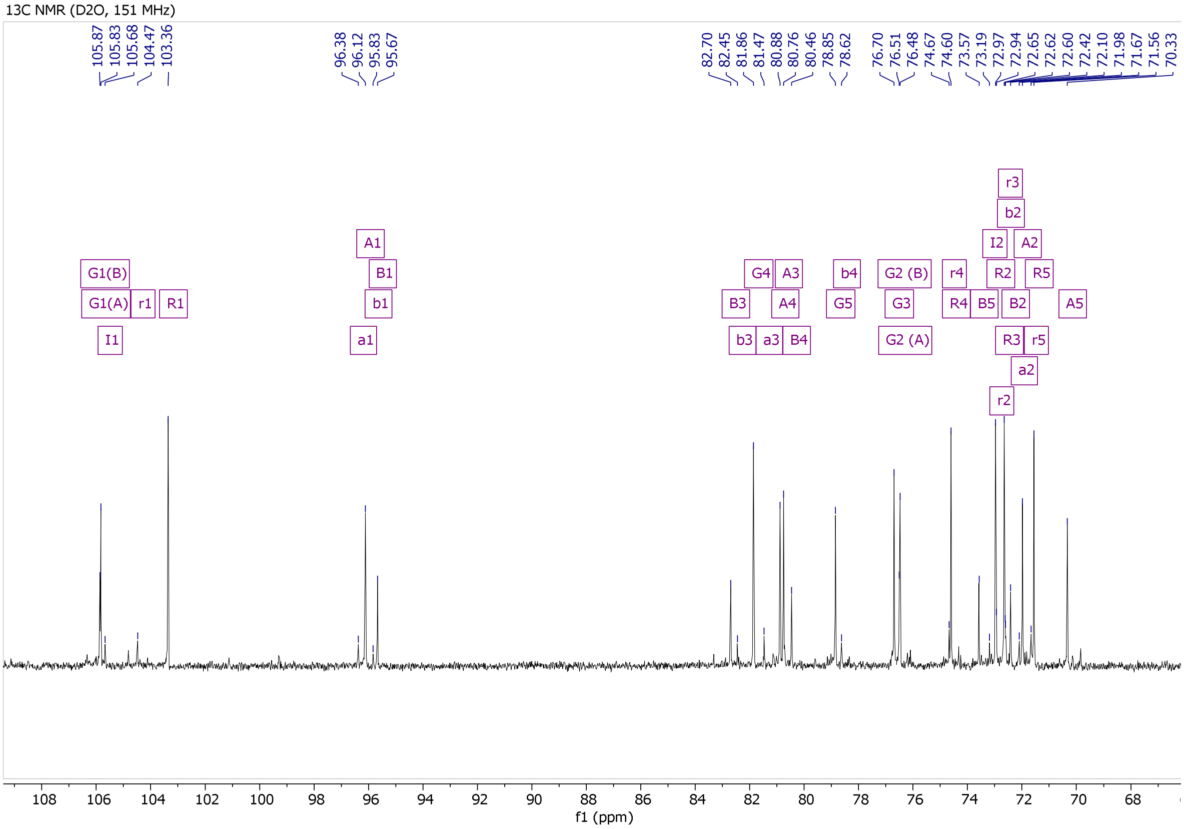

Figure S11. ^{13}C-NMR of the purified trimer mixture containing Rha3S-GlcA-Rha3S and Rha3S-IdoA-Rha3S (ratio ~7:3) – Zoom into the carbohydrate region

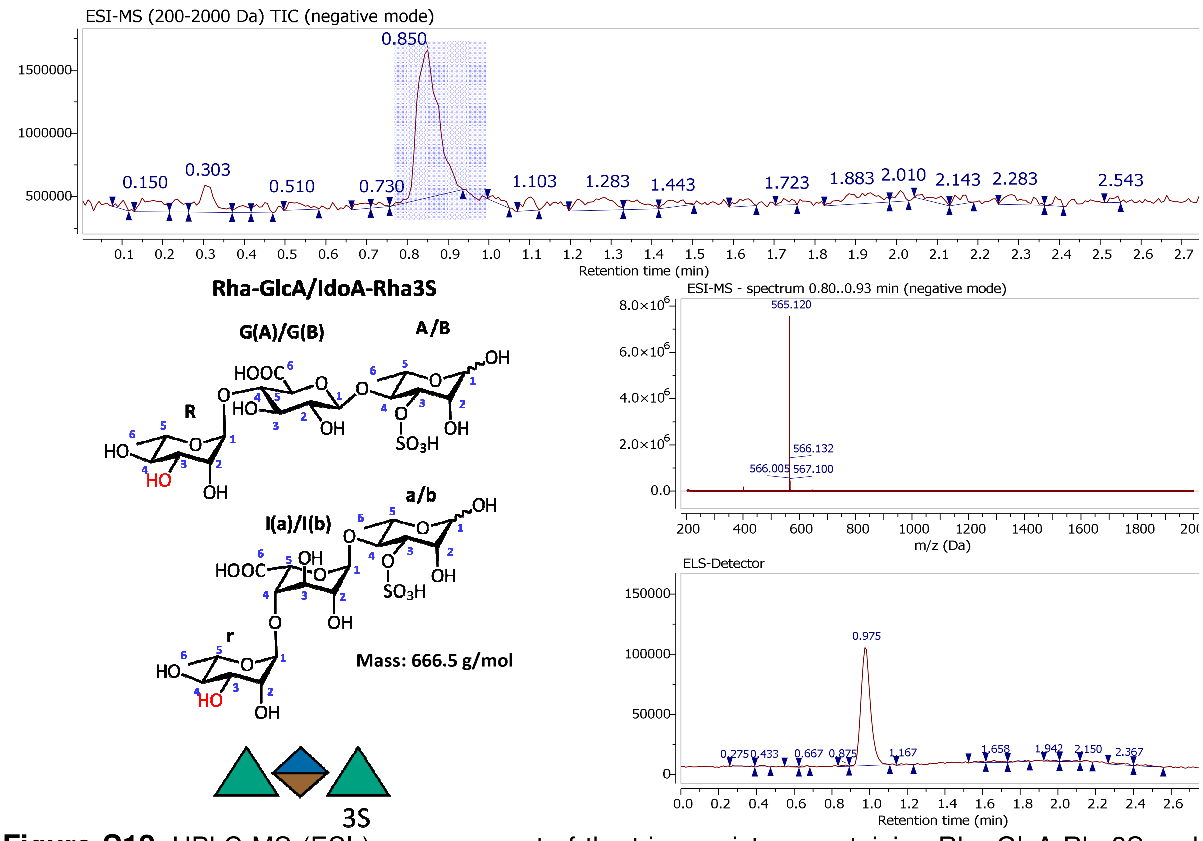

Figure S12. HPLC-MS (ESI-) measurement of the trimer mixture containing Rha-GlcA-Rha3S and Rha-IdoA-Rha3S (ratio ~7:3). Showing the [M-1] molecule peak of the compounds and no [M-80] signal, which was shown to be only present in oligosaccharide structures containing SO_3 on a non-reducing sugar.

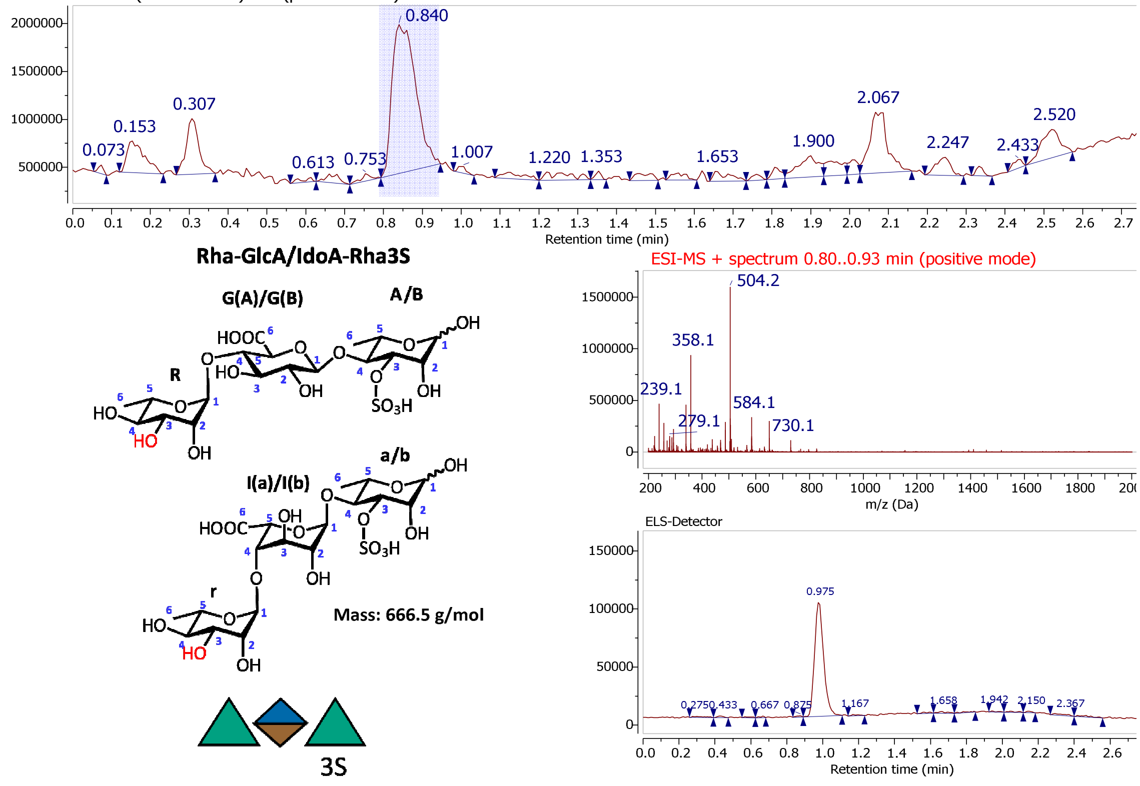

Figure S13. HPLC-MS (ESI+) measurement of the trimer mixture containing Rha-GlcA-Rha3S and Rha-IdoA-Rha3S (ratio ~7:3). Showing the [M+18] molecule peak of the compounds and no [M-80+18] signal, which was shown to be only present in oligosaccharide structures containing SO_3 on a non-reducing sugar.

Table S3a. NMR shifts of GlcA-dimer contained in a mixture of GlcA-Rha3S and IdoA-Rha3S (ratio ~7:3) originating from the digestion of Rha-GlcA-Rha3S with Enzyme P36_GH78. The chemical shifts are compared to the starting material. Indicative shifts of chemical shifts that support the claimed structure are marked in red. NMR spectra are shown in Figs. S14 to S17. ESI-MS measurements support the structure shown in Figs. S18 and S19). The shifts of the IdoA-component are shown in Table S3b.

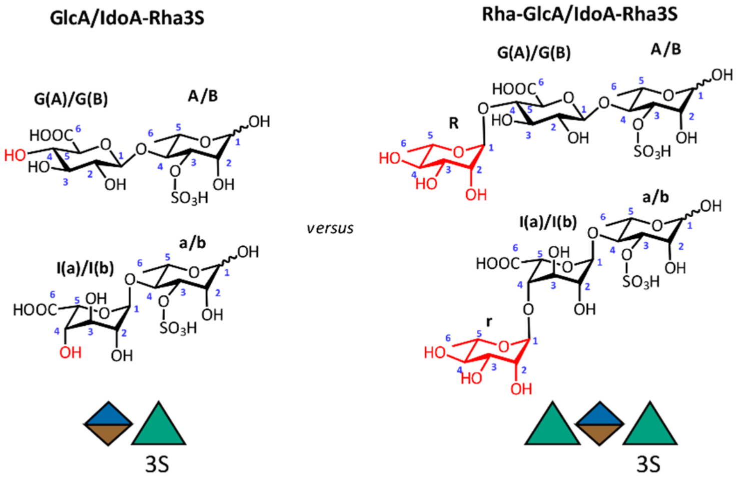

		^{1}H-Shifts (ppm)			^{13}C Shifts (ppm)		
		**GlcA Rha3S**	**Ref** Rha-GlcA-Rha3S	**Delta (A1=A1)**	**GlcA Rha3S**	**Ref** Rha-GlcA-Rha3S	**Delta (A1=A1)**
**A** α-Rhamnose	**1**	**5.13**	**5.08**	**0.00**	**93.46**	**96.12**	**0**
	**2**	4.27	4.22	0.00	69.36	71.98	0.04
	**3**	4.66	4.61	0.00	78.18	80.76	0.08
	**4**	3.82	3.75	0.02	78.27	80.88	0.05
	**5**	4.02	3.99	-0.02	67.76	70.33	0.09
	**6**	1.37	1.31	0.01	17.28	19.91	0.03
**B** β-Rhamnose	**1**	4.93	4.89	-0.01	93.04	95.67	0.03
	**2**	4.27	4.23	-0.01	69.8	72.42	0.04
	**3**	4.45	4.43	-0.03	80.14	82.7	0.1
	**4**	3.75	3.68	0.02	77.85	80.46	0.05
	**5**	3.55	3.52	-0.02	70.97	73.57	0.06
	**6**	1.37	1.32	0.00	17.28	19.91	0.03
**G(A)** Glucuronic acid (of α-anomer)	**1**	4.66	4.61	0.00	103.25	105.83	0.08
	**2**	3.34	3.33	-0.04	73.52	76.48	-0.3
	**3**	3.55	3.58	-0.08	75.44	76.7	1.4
	**4**	3.55	3.55	-0.05	71.89	81.86	-7.31
	**5**	3.71	3.7	-0.04	76.63	78.85	0.44
	**6**			-0.05	175.87	178.15	0.38
**G(B)** Glucuronic acid (of β-anomer)	**1**	4.66	4.61	0.00	103.25	105.83	0.08
	**2**	3.34	3.32	-0.03	73.52	76.51	-0.33
	**3**	3.55	3.58	-0.08	75.44	76.7	1.4
	**4**	3.55	3.53	-0.03	71.89	81.86	-7.31
	**5**	3.71	3.7	-0.04	76.63	78.85	0.44
	**6**			-0.05	175.87	178.15	0.38
		Referenced to HDO: 4.79 ppm					

Table S3b. NMR shifts of GlcA-dimer contained in a mixture of GlcA-Rha3S and IdoA-Rha3S (ratio ~7:3) originating from the digestion of Rha-GlcA-Rha3S with Enzyme P36_GH78. The chemical shifts are compared to the starting material NMR spectra are shown in Figs. S14 to S17. ESI-MS measurements support the structure shown in Figs. S18 and S19). The shifts of the GlcA-component are shown in Table S3a. Due to the very low concentration of the sample, complete assignment of the minor IdoA component was not possible for a significant amount of atoms.

		^{1}H-Shifts (ppm)			^{13}C Shifts (ppm)		
		**Rha-IdoA Rha3S**	**Ref** Rha3S-IdoA-Rha3S	**Delta (A1=A1)**	**Rha-IdoA Rha3S**	**Ref** Rha3S-IdoA-Rha3S	**Delta (A1=A1)**
**a** α-Rhamnose	**1**	93.68	96.38	0	93.68	96.38	0
	**2**	69.36	72.1	-0.04	69.36	72.1	-0.04
	**3**	78.67	81.47	-0.1	78.67	81.47	-0.1
	**4**	78.83			78.83		
	**5**	*			*		
	**6**	17.05	19.7	0.05	17.05	19.7	0.05
**b** β-Rhamnose	**1**	93.15	95.83	0.02	93.15	95.83	0.02
	**2**	*	72.6		*	72.6	
	**3**	80.69			80.69		
	**4**	*	78.62		*	78.62	
	**5**	*			*		
	**6**	17.05	19.7	0.05	17.05	19.7	0.05
**I(A)** Iduronic acid (of α-anomer)	**1**	102.81	105.68	-0.17	102.81	105.68	-0.17
	**2**	70.14	73.19	-0.35	70.14	73.19	-0.35
	**3**	76.73			76.73		
	**4**	71.51		74.21	71.51		
	**5**	*			*		
	**6**	*	177.25		*	177.25	
**I(B)** Iduronic acid (of β-anomer)	**1**	102.81	105.68	-0.17	102.81	105.68	-0.17
	**2**	70.14	73.19	-0.35	70.14	73.19	-0.35
	**3**	76.73			76.73		
	**4**	71.51		74.21	71.51		
	**5**	*			*		
	**6**	*	177.25		*	177.25	
***… unambigous assignment not possible**		*Referenced to HDO: 4.79 ppm*					

1H NMR (D2O, 600.15 MHz)

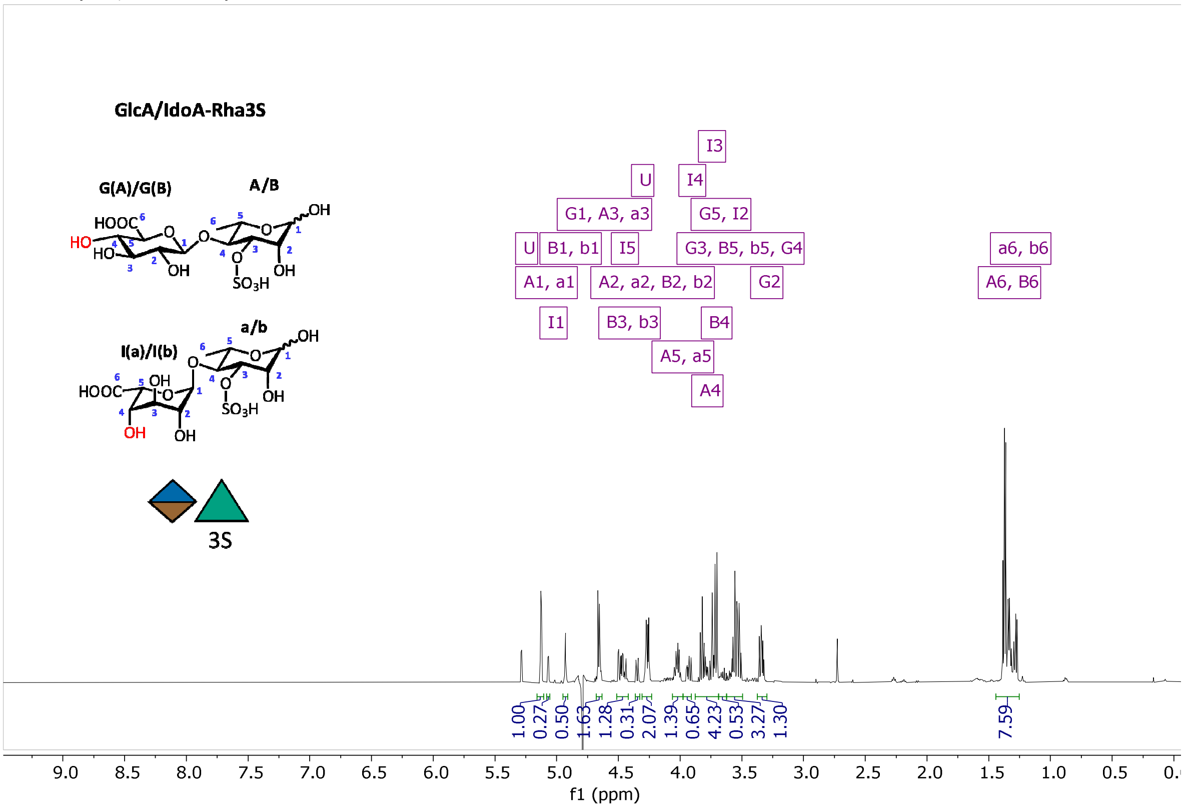

Figure S14. ^{1}H-NMR (with water suppression) of the dimer mixture containing GlcA-Rha3S and IdoA-Rha3S (ratio ~7:3) together with an unidentified substance (U) – Full View

1H NMR (D2O, 600.15 MHz)

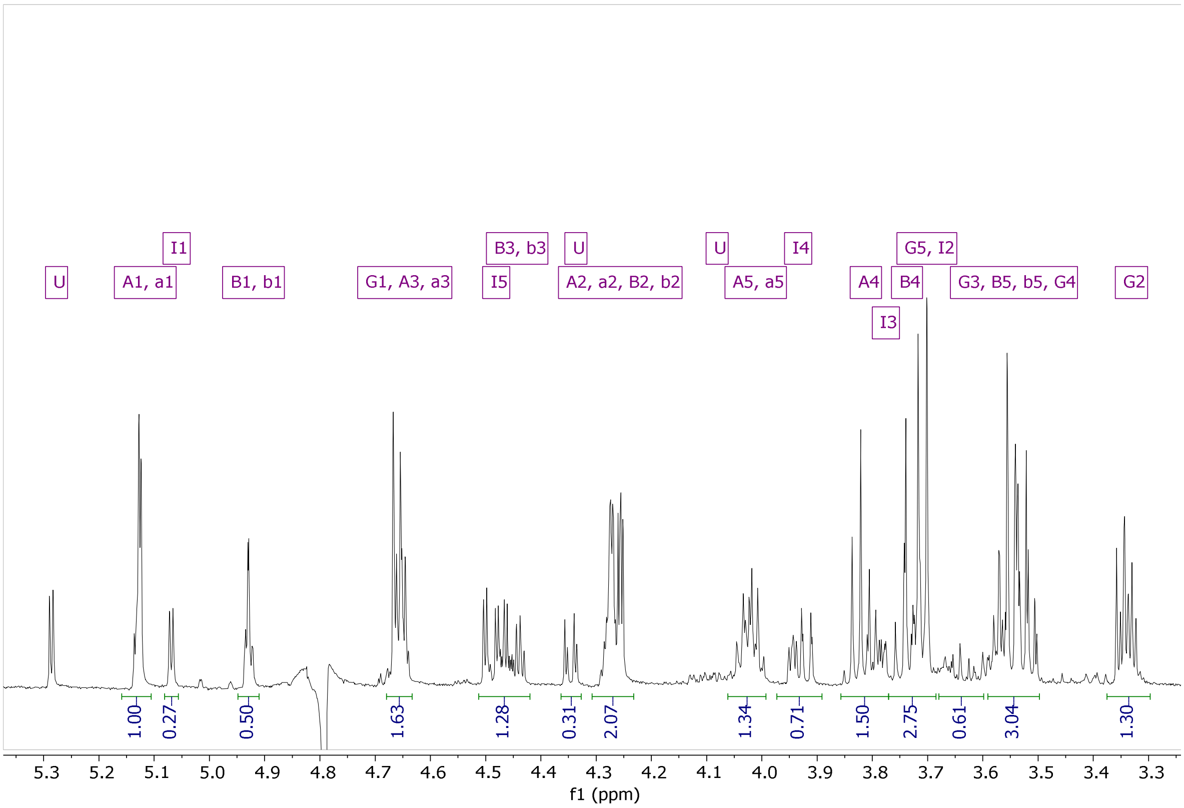

Figure S15. ^{1}H-NMR (with water suppression) of the dimer mixture containing GlcA-Rha3S and IdoA-Rha3S (ratio ~7:3) together with an unidentified substance (U) – Zoom into the carbohydrate region

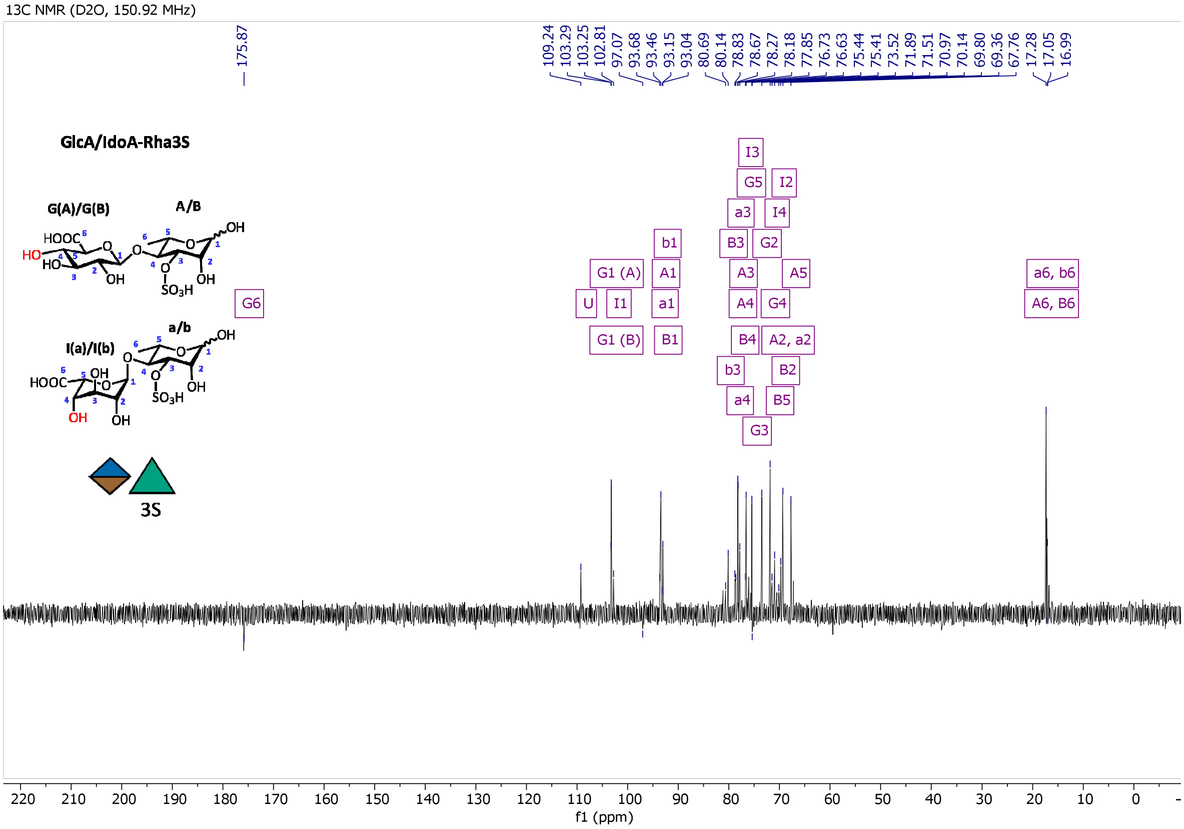

Figure S16. ^{13}C-NMR of the dimer mixture containing GlcA-Rha3S and IdoA-Rha3S (ratio ~7:3) together with an unidentified substance (U) – FullView

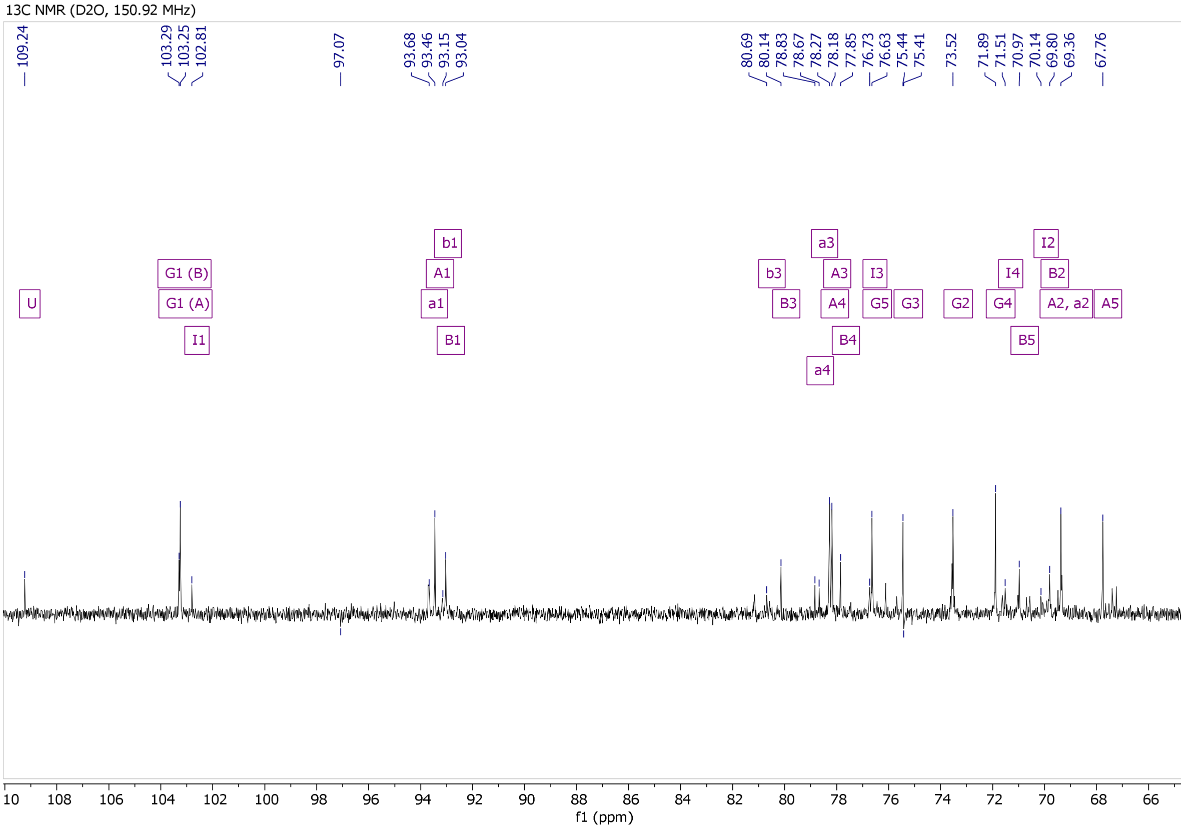

Figure S17. ^{13}C-NMR of the dimer mixture containing GlcA-Rha3S and IdoA-Rha3S (ratio ~7:3) together with an unidentified substance (U) – Zoom into the carbohydrate region

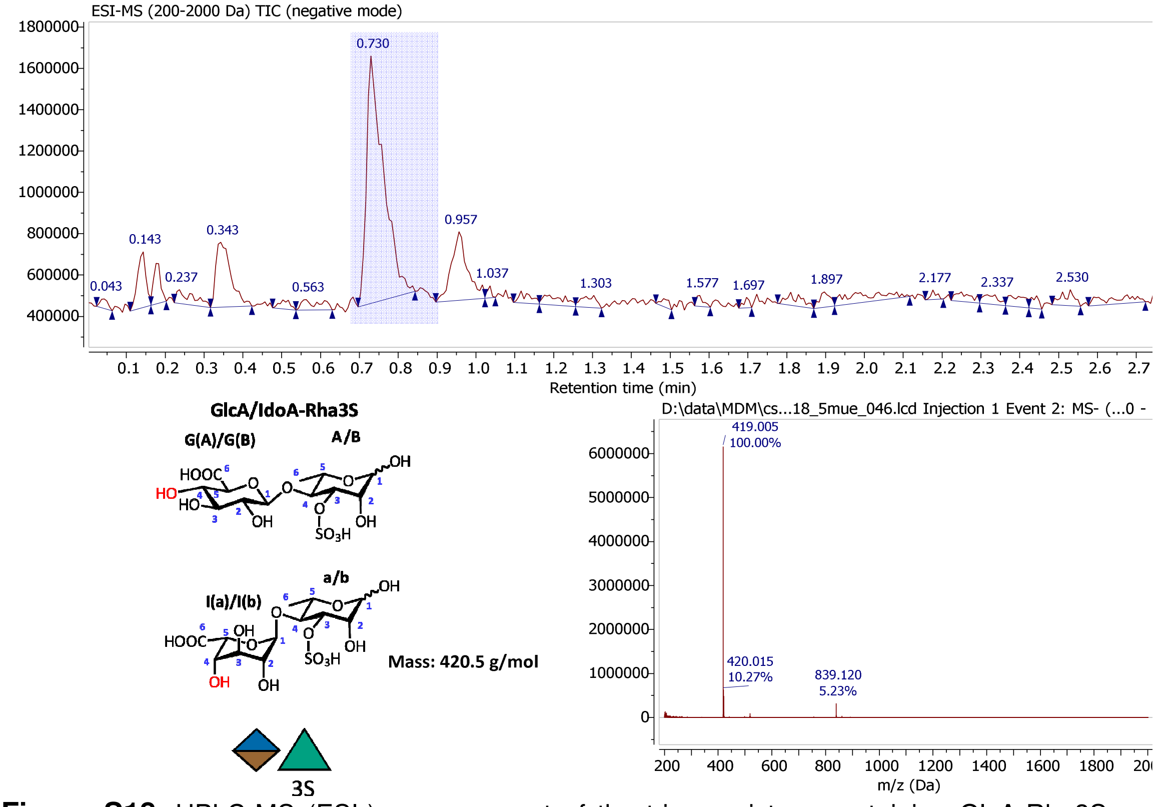

Figure S18. HPLC-MS (ESI-) measurement of the trimer mixture containing GlcA-Rha3S and IdoA-Rha3S (ratio ~7:3). Showing the [M-1] molecule peak of the compounds

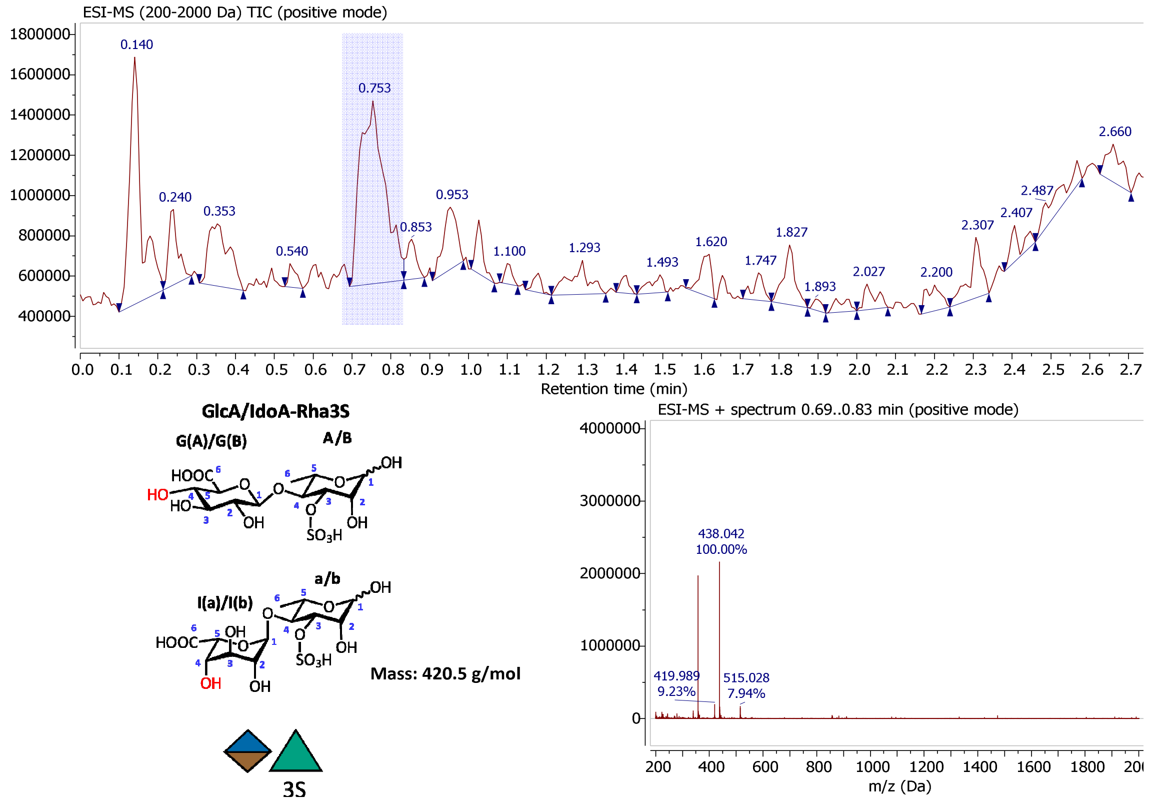

Figure S19. HPLC-MS (ESI+) measurement of the trimer mixture containing GlcA-Rha3S and IdoA-Rha3S (ratio ~7:3). Showing the [M+18] molecule peak of the compounds.

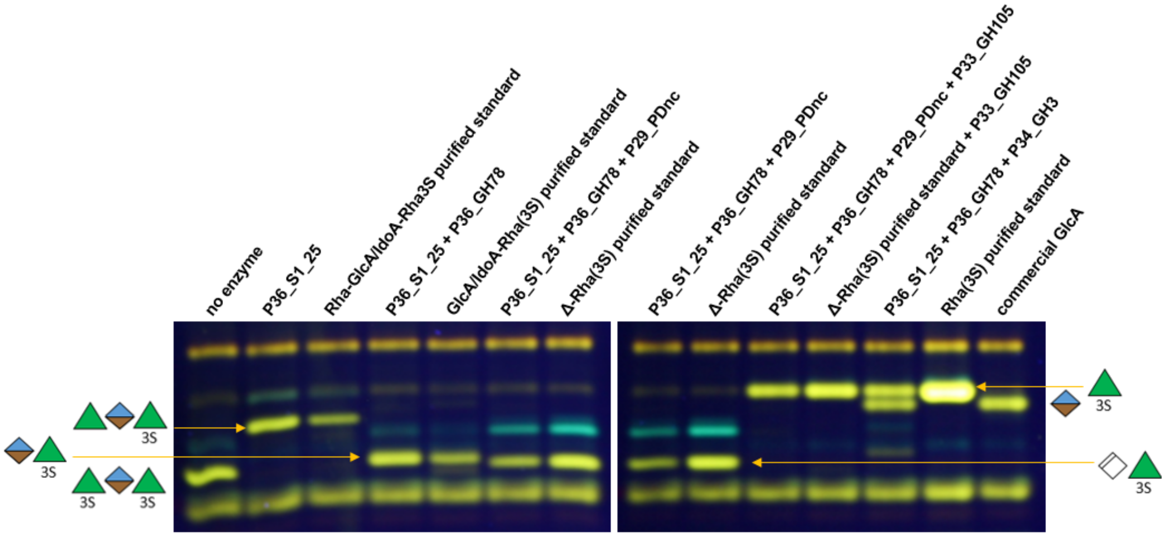

Figure S20. Complete-Gel FACE-analysis of the alternative ulvan degradation pathway of *Formosa agariphila*. In reactions containing P29_PDnc the other enzymes were heat-inactivated before addition of P29_PDnc to prevent a degradation of the dehydratase product by P33_GH105. All used products and standards (expect GlcA) have been isolated and confirmed by MS and NMR measurement. The standard for GlcA was obtained from Roth. All products represent the mixture of both oligomers containing one of the epimers GlcA or IdoA. The ratio between GlcA- and IdoA-containing oligomers is ~70:30 (10, 18).

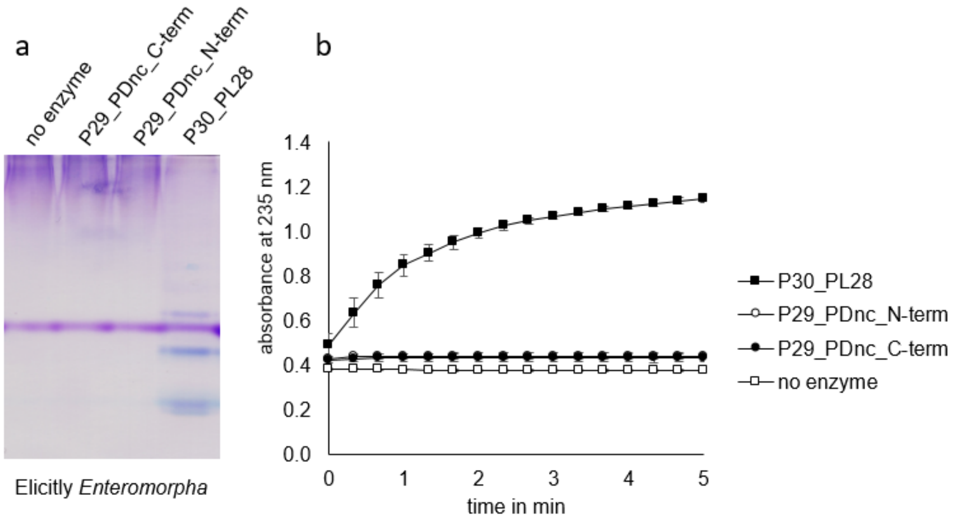

Figure S21. Analysis of lyase activity of P29_PDnc on commercial ulvan (Elicityl, France) from *Enteromorpha* sp. (left) C-PAGE analysis and (right) lyase assay. Polymeric ulvan from seven different sources has been incubated with both P29_PDnc variants with N-terminal or C-terminal His-tag and P30_PL28 as positive control or without enzymes as negative control. We compared two commercially available ulvans from Elicityl extracted from *Enteromorpha* sp. (S20) or *Ulva* sp. (S21), and five self-isolated ulvans from "kulau sea lettuce" containing *Ulva* spp. from Spain (S23), and from self-collected *Ulva* sp. from Lubmin (Baltic Sea) (S22), France (Atlantic Ocean) (S24, S25) and Helgoland (North Sea) (S26) (see the following Fig. S21-S26).

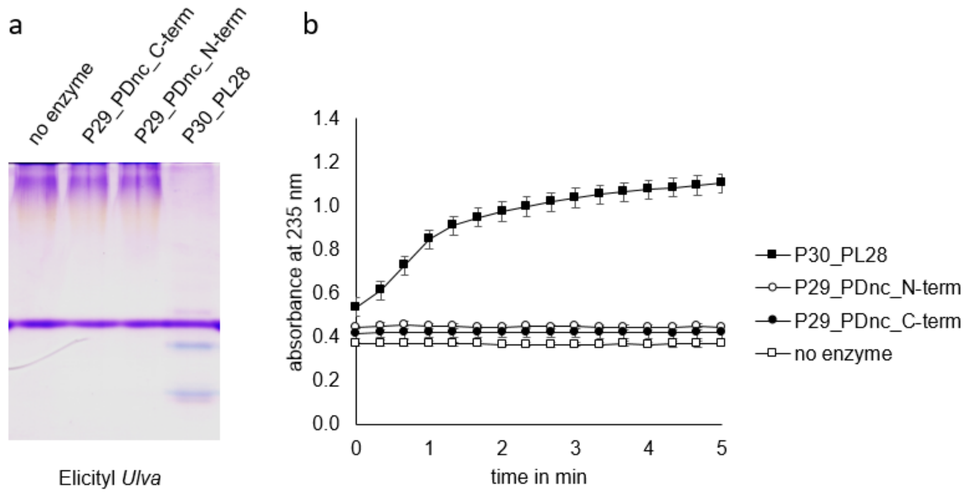

Figure S22. Analysis of lyase activity of P29_PDnc on commercial ulvan (Elicityl, France) from *Ulva sp*. (a) C-PAGE analysis and (b) lyase assay. Polymeric ulvan has been incubated with both P29_PDnc variants with *N*-terminal or *C*-terminal His-tag and P30_PL28 as positive control or without enzymes as negative control.

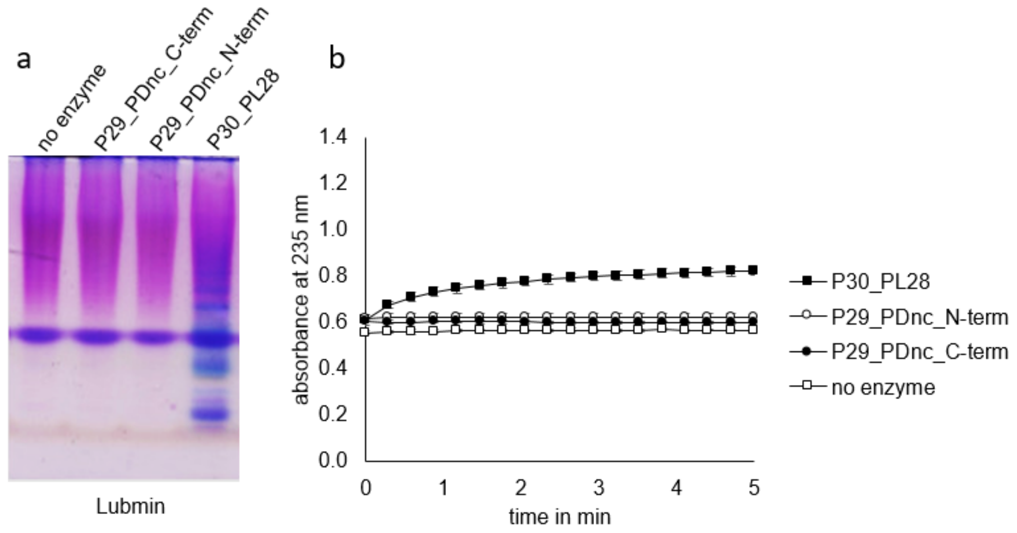

Figure S23. Analysis of lyase activity of P29_PDnc on self-isolated ulvan from *Ulva* sp. from Lubmin (Baltic Sea). (a) C-PAGE analysis and (b) lyase assay. Polymeric ulvan has been incubated with both P29_PDnc variants with *N*-terminal or *C*-terminal His-tag and P30_PL28 as positive control or without enzymes as negative control.

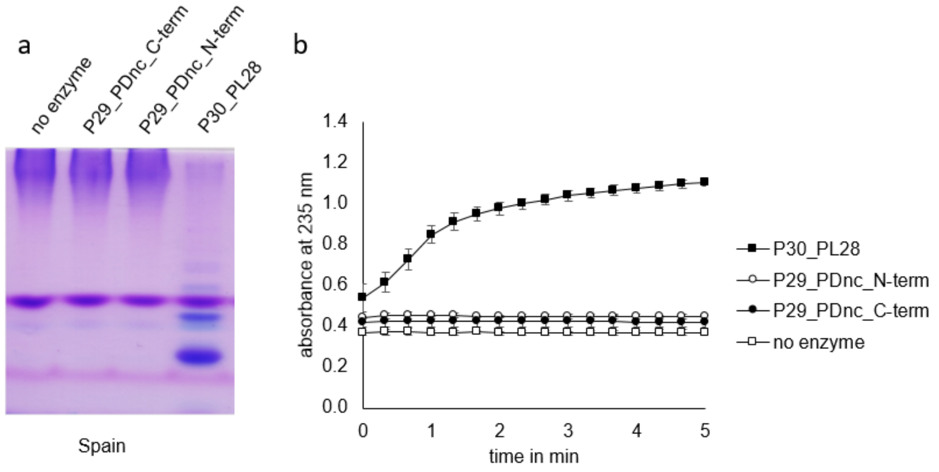

Figure S24. Analysis of lyase activity of P29_PDnc on self-isolated ulvan from *Ulva* sp. from Spain. (a) C-PAGE analysis and (b) lyase assay. Polymeric ulvan has been incubated with both P29_PDnc variants with *N*-terminal or *C*-terminal His-tag and P30_PL28 as positive control or without enzymes as negative control.

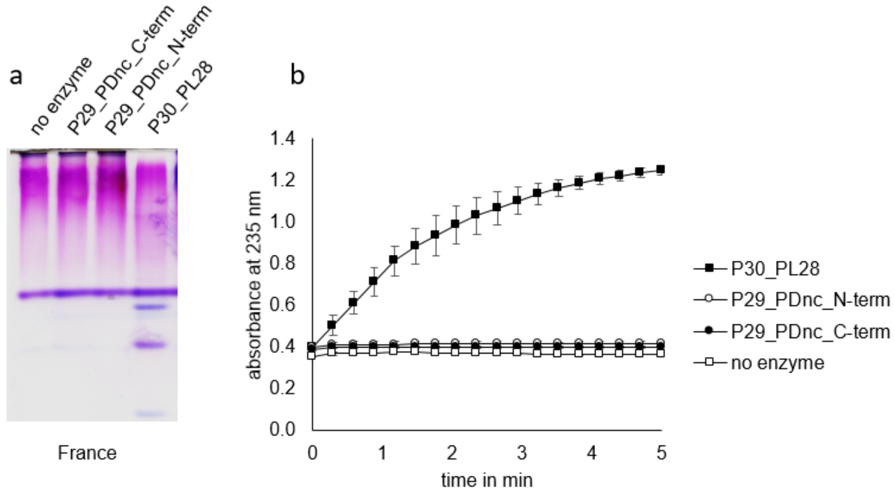

Figure S25. Analysis of lyase activity of P29_PDnc on self-isolated ulvan from self-collected *Ulva* sp. from France (Atlantic Ocean). (a) C-PAGE analysis and (b) lyase assay. Polymeric ulvan has been incubated with both P29_PDnc variants with *N*-terminal or *C*-terminal His-tag and P30_PL28 as positive control or without enzymes as negative control.

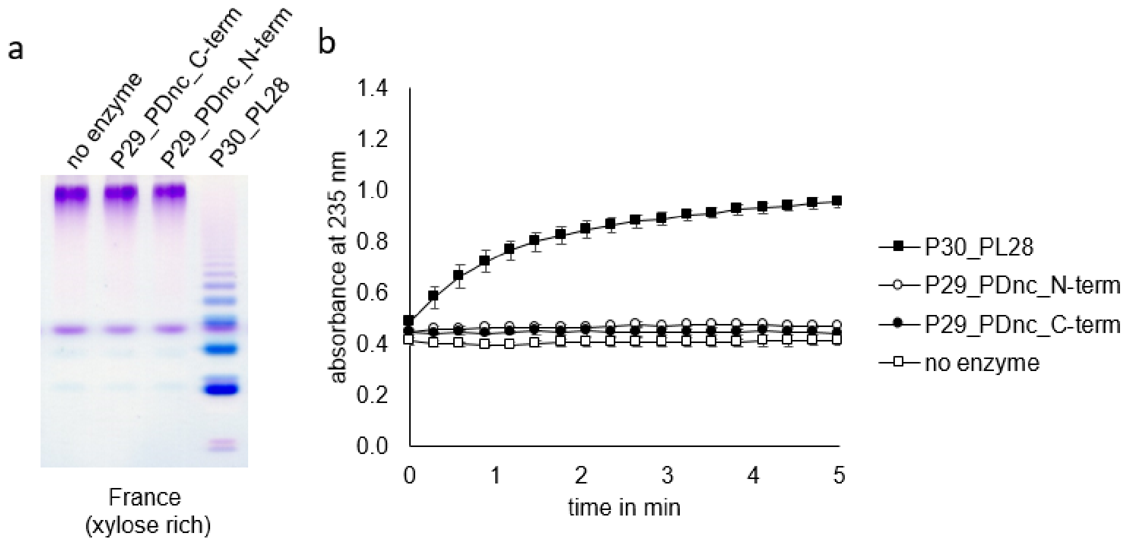

Figure S26. Analysis of lyase activity of P29_PDnc on self-isolated xylose-rich ulvan from self-collected *Ulva* sp. from France (Atlantic Ocean). (a) C-PAGE analysis and (b) lyase assay. Polymeric ulvan has been incubated with both P29_PDnc variants with *N*-terminal or *C*-terminal His-tag and P30_PL28 as positive control or without enzymes as negative control. Figure S26 shows the same data as in Figures 3A and 3B to allow a direct comparison with Figures 3C and 3D in the main publication as well as with Figure S27

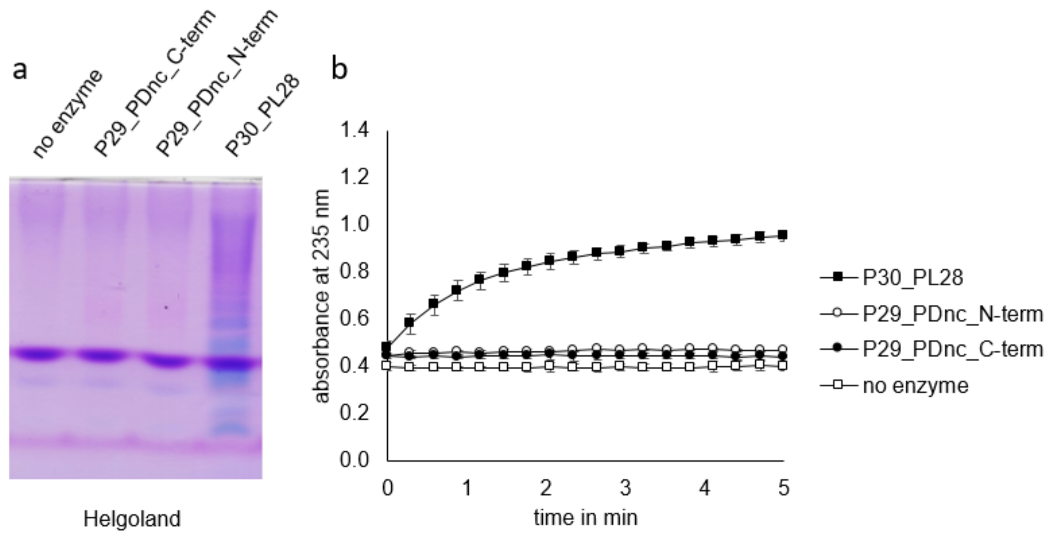

Figure S27. Analysis of lyase activity of P29_PDnc on self-isolated ulvan from self-collected *Ulva* sp. from Helgoland. (a) C-PAGE analysis and (b) lyase assay. Polymeric ulvan has been incubated with both P29_PDnc variants with *N*-terminal or *C*-terminal His-tag and P30_PL28 as positive control or without enzymes as negative control.

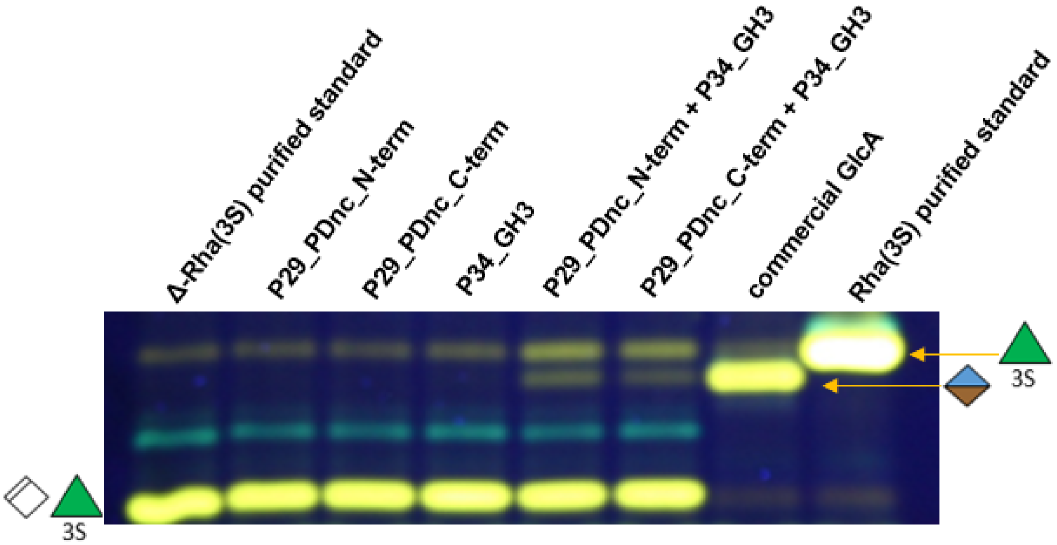

Figure S28. Hydration of Δ-Rha3S using P29_PDnc + P34_GH3 with FACE-analysis. P29_PDnc and P34_GH3 have been incubated on the disaccharide Δ-Rha3S leading to the formation of GlcA or IdoA and Rha3S in monomeric form by shifting the chemical equilibrium of the dehydration step by P29_PDnc to the educt by degrading the produced disaccharide GlcA/IdoA with P34_GH3.

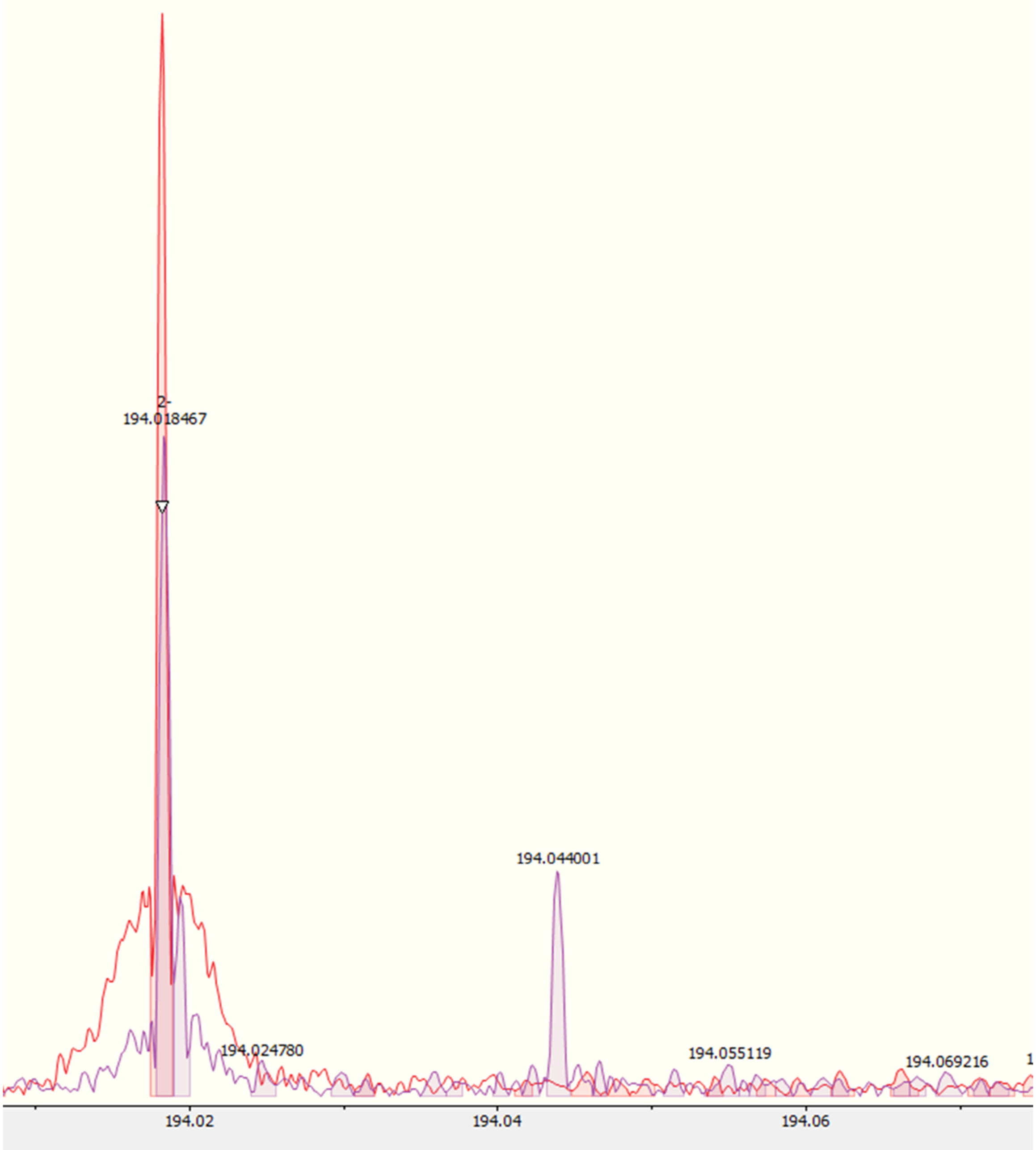

Figure S29. Mass spectrum for the hydration of Δ-Rha3S using P29_PDnc + P34_GH3. P29_PDnc and P34_GH3 were incubated on the disaccharide Δ-Rha3S leading to the formation of GlcA or IdoA and Rha3S in monomeric form by shifting the chemical equilibrium of the dehydration step by P29_PDnc to the educt by degrading the produced disaccharide GlcA/IdoA with P34_GH3. The mass spectrum shows the [M-H]- mass of glucuronic acid +1 (194.044001). The red spectrum represents the negative control without enzymes (glucuronic acid peak is missing here) while the purple spectrum represents the reaction with enzymes. The left large peak is not shown in its complete height to remain clarity of the purple reaction peak. The y-axis (intensity) is not shown in this image section.

Table S4. Primer list.

Plasmid	Gene BN863_*(protein abbreviation)	Forward (5'->3')	Reverse (5'->3')
pBAD-FGE	*30240	AACAGGAGGAATTAACCATGATGTCTTTAAAAGAAAACTACATAACTACG	TGGTCGACGGCGCTATTTTATTTTACTAATCTAATTCCTGAAAATTG
pET28-#29	*22180 (P29_PDnc)	AGCGGCCTGGTGCCGCGCGGCAGCCATATGTGCTCTAGTTTCCCCGAAC	GTGGTGGTGGTGGTGCTCGAGTGCGGCCGCTTAAACTACAGAAACAATTAGCACC
pET28-#30	*22190 (P30_PL28)	TCATCACAGCAGCGGCCTGGTGCCGCGCGGCAGCCATATGCAAACCGCGCCCGATGAAG	CAGTGGTGGTGGTGGTGGTGCTCGAGTGCGGCCGCTTACAGAGACTCAACAGCAATAGC
pET28-#33	*22220 (P33_GH105)	TCACAGCAGCGGCCTGGTGCCGCGCGGCAGCCATATGCAAAAAGGGCTAAACCATTC	TCAGTGGTGGTGGTGGTGGTGCTCGAGTGCGGCCGCTTACTCTTCCAGTTTTAAAACTTC
pET28-#34	*22230 (P34_GH3)	AGCGGCCTGGTGCCGCGCGGCAGCCATATGCAACGAACCTATACCCAGTC	GTGGTGGTGGTGGTGCTCGAGTGCGGCCGCTTATTTTTTTAATTGAAATGAAGTTTT
pFA34	*22250 (P36_GH78)	TTTTTTCTCGAGGAGTTTAGAGCTGGACCGTCTGA	TTTTTTCTGCAGTTAAAATGAGTTAATTTTTAATTCAATAATAT

Table S5. Protein concentrations and corresponding biocatalysis concentrations while 15 µg/mL of the proteins were used in the reaction.

Protein	Concentration [µg/ml]	Concentration [µM]	Concentration in biocatalyis reaction [µM]
P30_GH105	15958	362.68	0.34
P36_S1_25	159	3.31	0.31
P36_GH78	1106	13.33	0.18
P29_PDnc_N-term	909	13.98	0.23
P29_PDnc_C-term	637	9.23	0.21
P34_GH3	3656	44.8	0.18
P29_D248N	859	13.22	0.23
P29_E254Q	678	10.43	0.23
P29_E255Q	1285	19.77	0.23
P29_D269N	1466	22.55	0.23
P29_D290N	655	10.08	0.23

P29_R300E	1246	19.17	0.23
P29_K303I	1075	16.54	0.23
P29_Y306F	967	15.02	0.23

Table S6. Nucleotide sequence (codon-optimized) encoded in the enzymes study

>**P36_S1_25**	CAGACCGTGAAGAAGGAGAAGCCGAACATCATTTTCATCCTGACCGACGAT<br>CAGCGTTTCGACGCGATTGGTTATGCGGGTAATAAGTTCGTGAACACCCCG<br>GAAATGGATAAGCTGGCGCAGCAAGGCACCTACTTTGACCACGCGATCGTT<br>ACCACCCCGATTTGCGCGGCGAGCCGTGCGAGCCTGTGGACCGGCCTGC<br>ATGAGCGTAGCCACAACTTCAACTTTCAGACCGGTAACGTGCGTGAGGAAT<br>ATATGAACAACGCGTACCCGAAGCTGCTGAAAAACAACGGTTACTATACCG<br>GTTTCTATGGCAAATACGGTGTTCGTTATGACAACCTGGAAAGCCAATTCGA<br>CGAGTTTGAAAGCTATGATCGTAACAACCGTTACAAAGATAAGCGTGGCTA<br>CTATTACAAGACCATCAACAACGACACCGTGCACCTGACCCGTTACACCGG<br>TCAGCAAGCGATCGACTTCATTGATAAAAACGCGACCAACACCCAGCCGTT<br>CATGCTGAGCCTGAGCTTTAGCGCGCCGCACGCGCATGATGGTGCGCCGG<br>AACAGTATTTTTGGCAAACCACCACCGACGCGCTGCTGCAAGATACCACCC<br>TGCCGGGTCCGGACCTGGCGGATGAGAAGTACTTCCTGGCGCAGCCGCAA<br>GCGGTTCGTGACGGTTTTAACCGTCTGCGTTGGACCTGGCGTTATGACGAT<br>CCGGAGAAGTACCAGCACAGCCTGAAAGGCTATTACCGTATGATCAGCGG<br>TATTGACCTGGAAATCAAGAAAATTCGTGATAAACTGAAGGAGAAAGGTGT<br>GGACAAAAACACCGTGATCATTGTTATGGGCGATAACGGTTATTTCCTGGG<br>CGAACGTCAACTGGCGGGCAAGTGGCTGATGTACGACAACAGCATCCGTG<br>TGCCGCTGATTGTTTTTGATCCGCGTGTTAACAAACACCAGGACATCAGCG<br>AGATGGTGCTGAACATCGACGTTACCCAAACCATTGCGGATCTGGCGGGC<br>GTGAAGGCGCCGGAAAGCTGGCAGGGCAAGAGCCTGCTGCCGCTGGTTA<br>AACAAGAAACCAGCACCATCAGCCGTGATACCATCCTGATTGAGCACCTGT<br>GGGACTTCGAAAACATTCCGCCGAGCGAGGGCGTGCGTACCGAGGAATGG<br>AAGTATTTTCGTTACGTTAACGATAAAACCATCGAGGAACTGTATAACATTA<br>AGAAAGACCCGAAAGAAATCAACAACCTGATTGGTAAGAAAAAGTACCAGA<br>ACGTGGCGAAGGCGCTGCGTGAAAAACTGGACGAACTGATTGCGAAAAAT<br>AGCGACGAATTCCGTTAA
